# Understanding belief in political statements using a model-driven experimental approach: a registered report

**DOI:** 10.1038/s41598-023-47939-3

**Published:** 2023-12-01

**Authors:** Agustín Perez Santangelo, Guillermo Solovey

**Affiliations:** 1 Instituto de Investigación en Ciencias de la Computación, Universidad de Buenos Aires (UBA), Consejo Nacional de Investigaciones Científicas y Técnicas (CONICET), C1428EGA Buenos Aires, Argentina; 2 Laboratorio de Neurociencia, CONICET, Universidad Torcuato Di Tella, C1428BIJ Buenos Aires, Argentina; 3 Instituto de CálculoFacultad de Ciencias Exactas y Naturales, UBA-CONICET, Buenos Aires, Argentina

**Keywords:** Human behaviour, Computational models

## Abstract

**Abstract:**

Misinformation harms society by affecting citizens' beliefs and behaviour. Recent research has shown that partisanship and cognitive reflection (i.e. engaging in analytical thinking) play key roles in the acceptance of misinformation. However, the relative importance of these factors remains a topic of ongoing debate. In this registered study, we tested four hypotheses on the relationship between each factor and the belief in statements made by Argentine politicians. Participants (N = 1353) classified fact-checked political statements as true or false, completed a cognitive reflection test, and reported their voting preferences. Using Signal Detection Theory and Bayesian modeling, we found a reliable positive association between political concordance and overall belief in a statement (median = 0.663, CI95 = [0.640, 0.685]), a reliable positive association between cognitive reflection and scepticism (median = 0.039, CI95 = [0.006, 0.072]), a positive but unreliable association between cognitive reflection and truth discernment (median = 0.016, CI95 = [− 0.015, 0.046]) and a positive but unreliable association between cognitive reflection and partisan bias (median = 0.016, CI95 = [− 0.006, 0.037]). Our results highlight the need to further investigate the relationship between cognitive reflection and partisanship in different contexts and formats.

**Protocol registration:**

The stage 1 protocol for this Registered Report was accepted in principle on 22 August 2022. The protocol, as accepted by the journal, can be found at: https://doi.org/10.17605/OSF.IO/EBRGC.

## Introduction

Misinformation is part of the world we live in, spreading through online messages, news headlines, published articles or disseminated by political leaders^1^. Defined as any statement that contradicts or distorts the best available evidence about verifiable facts^2,3^, misinformation has the potential to damage individuals and society at large. For instance, it has been associated with distrust in vaccination^2–4^, disbelief in climate change^5^ and unwillingness to wear masks during the COVID-19 pandemic^6^.

Political misinformation is especially concerning^7^. A study of the consumption of *fake news* during three-months prior to the US 2016 presidential election found that, on average, every US citizen encountered one to three “fake news” during that period^8^. In addition, false news spreads faster and reaches more people than true stories, particularly for politics^9^. Misinformation can fuel hostile behaviour^10^, interplay with political polarisation^11^, and interfere with the ability of individuals to make decisions guided by reliable knowledge—a hallmark of the democratic process—^12–14^. By contrast, well-informed individuals are more willing to participate in politics, are more politically tolerant, and hold more stable opinions over time^15^.

Focused on the online spread of fake news, research has examined the cognitive processes that underlie belief in misinformation^11,16^, informing strategies to minimise its dissemination^11,17–19^. However, the relative importance of analytical thinking and political motivations to explain belief in misinformation remains controversial^11,16,20,21^.

Belief in misinformation has been mainly attributed to an overreliance on intuitions, exposing individuals’ inability to reflect about the veracity of information^16^. Consistent with dual process theories of cognition^22,23^, this account suggests that the propensity to engage in analytical thinking and override wrong intuitions, known as cognitive reflection, leads to more accurate beliefs. In fact, more reflective people are better at discerning between true and false information, i.e. those with higher cognitive reflection scores are also more likely to believe in true headlines and discard fake news, regardless of whether the information aligns with their political ideology^16,24^. In this line, a recent study suggests that there is a causal link between deliberation and truth discernment^25^.

Also, information processing can be influenced by partisanship, i.e., loyalty to an ideological group or identification with a political party^26–28^. In particular, people are more likely to believe favourable information and discard unfavourable information about the party or leader they support, regardless of whether the information is true or false^16,20,27,29–31^. A recent review of 14 studies found that political concordance is a major factor to explain belief in fake news^16^.

On top of that, in some circumstances having more analytical thinking skills is associated with more biased beliefs^26,32,33^—but see^34,35^. In this line, more reflective individuals may spend more cognitive resources trying to convince themselves that their viewpoint is correct, amplifying partisan biases^33,36,37^. However, in the context of fake news detection, the interaction between cognitive reflection and partisanship is inconclusive^29^. It is unclear why the amplification of polarised beliefs among more reflective individuals holds in some contexts while it does not seem relevant in fake news detection.

Signal Detection Theory (SDT^38^) is a useful framework to understand belief in misinformation^29^. A cornerstone of SDT is that in binary decision making tasks there are two independent aspects of the underlying cognitive process: discrimination accuracy and response bias. Discrimination accuracy, termed truth discernment ability in misinformation studies^16^ or *d′* within SDT, is the ability to correctly categorize true and false information. Response bias, or *c* within SDT, refers to the tendency to claim that a piece of information is true or false. The independence between these two aspects implies that, for instance, having a large truth discernment ability entails neither a low probability of believing a piece of information is true (i.e., being overly sceptical) nor a low probability of believing it is false (i.e., being overly confident). This way, cognitive factors—such as partisanship and cognitive reflection—may be related to either, none, or both aspects of the detection process.

One limitation of previous research is that it focused on fake news, but misinformation spreads in different formats and media (e.g., TV shows, radio, newspapers). To understand belief in misinformation, an unexploited type of stimuli are false claims made by politicians. Nevertheless, the ability to determine the veracity of political leaders' discourse is critical, as it is known to directly influence public behaviour. For example, social distancing in Brazil was severely reduced right after the former president inaccurately minimized the mortality of COVID-19^39^. Moreover, using true/false political statements in experimental settings circumvents some disadvantages of prior work using real/fake social media headlines. While Facebook-like posts include the name of the media outlet (e.g. “thelastlineofdefense.org” for fake news and “The Washington Post” for real news^24^), statements do not. Therefore, the truthfulness of those claims cannot be inferred by simply assessing the credibility of the media outlet, which is a known proxy of the veracity of information^40,41^.

We will next present four research questions and theoretical support for our hypotheses, summarised in Table 1.

**Table 1:** Design table.

Question	Hypothesis	Sampling plan (e.g. power analysis)	Pre-registered analysis plan	Interpretation given to different outcomes
**RQ1**: Is the disposition to engage in cognitive reflection helpful to tell apart true from false political statements?	**H1**: Individuals' cognitive reflection score will have a positive association with their truth discernment ability, for any degree of numerical ability	N ≥ 1200 (see “Sampling plan” section) As we integrated all our hypotheses under the same model, this planned sample size applies to all RQs	We will extract the ${\delta }_{crt\_score}$ coefficient from the equal-variance Bayesian hierarchical Signal Detection Theory model, and assess whether it is reliably greater than 0, as described in the “Analysis plan” section (see Eq. 1) As we integrated all our hypotheses under the same model, this analysis plan applies to all RQs	If ${\delta }_{crt\_score}$ is reliably greater than 0, we will interpret this result as: higher cognitive reflection is associated with better truth discernment If it is reliably smaller than 0, we will interpret this result as: higher cognitive reflection is associated with worse truth discernment If it is not reliably different from 0, we will interpret this result as: there is not reliable evidence for an association between cognitive reflection and truth discernment
**RQ2**: Is partisanship associated with biased judgements about the veracity of political statements?	**H2**: Overall belief in political statements will increase with political concordance	–	We will extract the ${\lambda }_{pol\_concord}$ coefficient and assess whether it is reliably smaller than 0	If ${\lambda }_{pol\_concord}$ is reliably smaller than 0, we will interpret this result as: higher political concordance is associated with greater belief in that a statement is true, regardless of the actual veracity of that statement If it is reliably greater than 0, we will interpret this result as: higher political concordance is associated with less belief in that a statement is true, regardless of the actual veracity of that statement If it is not reliably different from 0, we will interpret this result as: there is not reliable evidence for an association between political concordance and belief in that a statement is true, regardless of the actual veracity of that statement
**RQ3**: Does cognitive reflection amplify the effect of political concordance?	**H3**: Individuals' cognitive reflection score will have a positive association with the difference between overall belief for concordant and discordant statements	–	We will extract the ${\lambda }_{pol\_concord:crt\_score}$ coefficient and assess whether it is reliably smaller than 0	If ${\lambda }_{pol\_concord:crt\_score}$ is reliably smaller than 0, we will interpret this result as: higher cognitive reflection is associated with greater ability to rationalize ideologically concordant information while dismissing discordant information If it is reliably greater than 0, we will interpret this result as: higher cognitive reflection is associated with lower ability to rationalize ideologically concordant information while dismissing discordant information If it is not reliably different from 0, we will interpret this result as: there is not reliable evidence for an association between cognitive reflection and the ability to rationalize ideologically concordant information while dismissing discordant information
**RQ4**: Is cognitive reflection associated with higher scepticism?	**H4**: Individuals' cognitive reflection score will have a negative association with overall belief	–	We will extract the ${\lambda }_{crt\_score}$ coefficient and assess whether it is reliably greater than 0	If ${\lambda }_{crt\_score}$ is reliably greater than 0, we will interpret this result as: higher cognitive reflection is associated with greater scepticism If it is reliably smaller than 0, we will interpret this result as: higher cognitive reflection is associated with less scepticism If it is not reliably different from 0, we will interpret this result as: there is no reliable evidence for an association between cognitive reflection and scepticism

### RQ1: Is the disposition to engage in cognitive reflection helpful to tell apart true from false political statements?

Dual-process theories of reasoning and decision making distinguish between intuitive processes (fast, impulsive and automatic) and reflective processes (which involve a more careful evaluation of the information)^22,23^. This is particularly relevant for politics. Given that political issues can elicit charged emotions, it is often hard to make political decisions through cold and dispassionate analysis^30,31^ that often require more cognitive effort than individuals may be willing to expend^36^.

This line of research suggests that the propensity to engage in analytical thinking, i.e. cognitive reflection, facilitates discerning between true and false information. The Cognitive Reflection Test (CRT) consists of a set of three problems introduced to measure the tendency to override intuitive but incorrect answers^42^. To illustrate, consider one item of the CRT: “A bat and a ball cost $1.10. The bat costs $1.00 more than the ball. How much does the ball cost?” Individuals that respond “10 cents”, which is incorrect albeit impulsive, find the problem easier than those that answer correctly. Those that give the correct response, normally also report to have considered the impulsive response first^42–44^. Further research showed that the score in the CRT varies substantially among individuals and it is a unique predictor of performance on heuristics-and-biases tasks^45^.

Applied to fake news, recent studies found that more reflective individuals are better at discerning fake news from real news^24,25,29,46,47^ (for a review, see^16^). Individuals with higher CRT scores are more likely to believe in veridical stories and recognize fake news headlines. Therefore, we hypothesise that this will hold true for judging the veracity of political statements. As some degree of numerical ability is required to solve the CRT problems^48^, we will statistically control for numeracy^49^.

**Figure 1:**
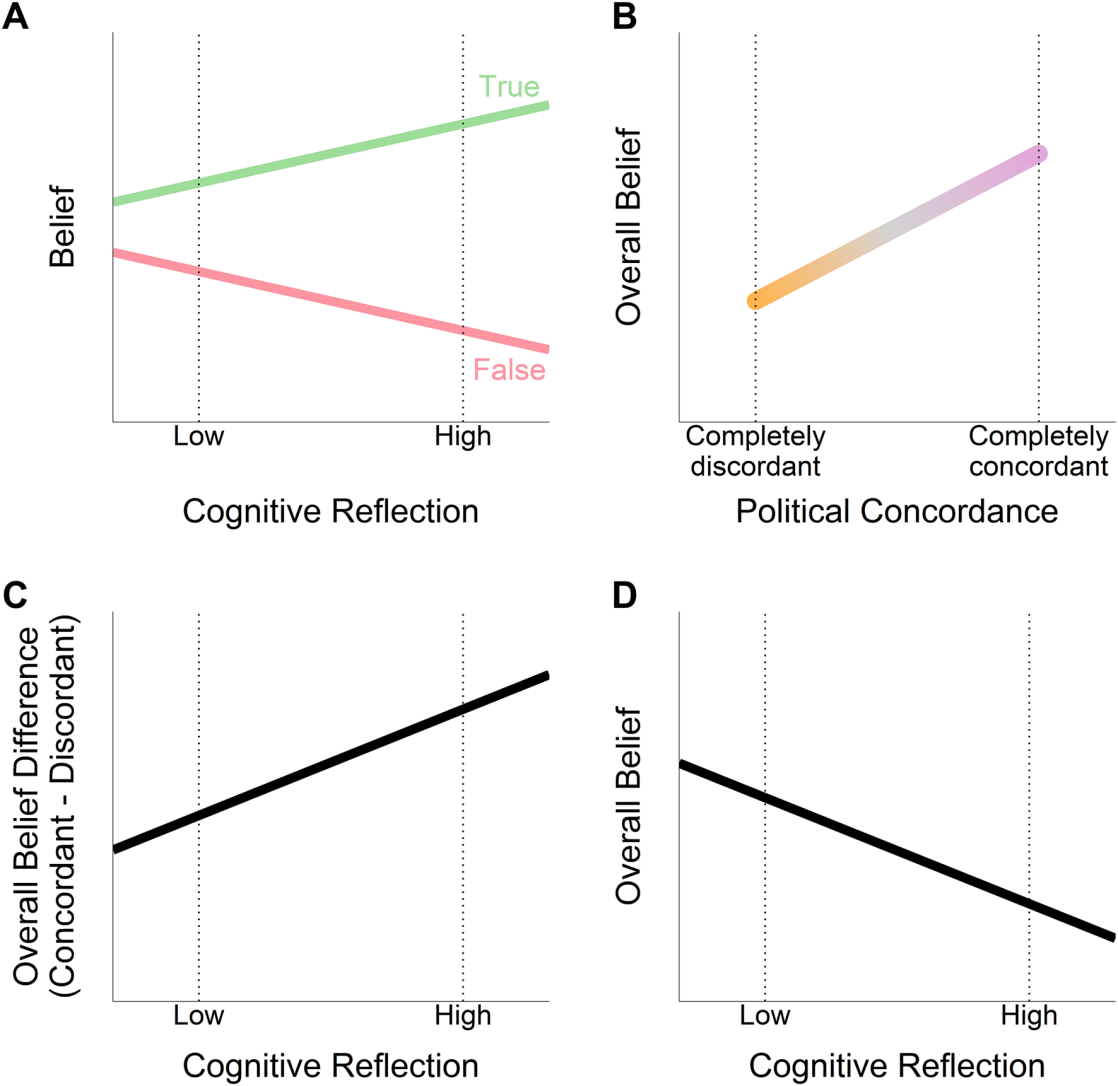
Hypotheses. Schematic representations of the relationship between variables targeted by our four hypotheses (see “Introduction” and Table 1). (**A**) Per hypothesis 1 (H1), the ability to discern true (green line) from false (red line) statements—truth discernment ability—will increase with higher cognitive reflection. That is, as cognitive reflection increases, the probability of saying that a statement is true—belief—will increase for true statements while it will also decrease for false statements. Note that the linear mapping depiction between belief and cognitive reflection is only for graphical simplicity; these relations need not be linear, just monotonical (reflected in our choice of a *probit* function to map z-scores to probability). (**B**) Per hypothesis 2 (H2), the propensity to say “true” regardless of the veracity of the statement—overall belief—will increase as the alignment between statement’s political valence and individual’s political profile (i.e., political concordance, from completely discordant [orange] to completely concordant [pink]) increases. Thus, overall belief for concordant statements will be higher than for discordant statements (partisan bias). (**C**) Per hypothesis 3 (H3), the difference between overall belief for concordant and discordant statements will increase with cognitive reflection. Thus, higher cognitive reflection will be associated with higher partisan bias on overall belief. (**D**) Per hypothesis 4 (H4), overall belief will decrease with cognitive reflection. In other words, scepticism (the complement of overall belief) will increase with cognitive reflection.

*H1: Individuals' cognitive reflection score will have a positive association with their truth discernment ability, for any degree of numerical ability (*Fig. 1A*).*

### RQ2: Is partisanship associated with biased judgements about the veracity of political statements?

Partisanship can influence opinions^50^, shape policy preferences^51,52^, distort interpretation of political facts^53,54^, alter vision^32,55^ and affect memory^56^.

In fake news detection tasks where participants have to decide whether a headline is true or false, partisanship is a strong predictor of the probability of “true” responses^16,27^. Using the terminology of Pennycook and Rand^16^, overall belief (the extent to which a statement is accepted as true) is larger for politically concordant than for discordant information. For example, a supporter of a political leader may uncritically believe fake news that favours said leader, i.e. politically concordant headlines, independently of the actual veracity of the information. This effect has been called the effect of political concordance on overall belief^16^ or, simply, partisan bias^29^.

It is unclear whether partisan biases are the result of motivated cognition or simply reflect optimal use of prior knowledge^57^. For instance, selective exposure to ideologically congruent news outlets may lead individuals to beliefs that, in turn, bias the evaluation of the veracity of new information. However, regardless of the underlying cognitive mechanisms, people are more likely to believe politically concordant headlines^16,27,29^.

Therefore, in line with previous findings, we predict evaluating political statements will follow the same pattern.

*H2: Overall belief in political statements will increase with political concordance (*Fig. 1B*).*

### RQ3: Does cognitive reflection amplify the effect of political concordance?

Early research in motivated cognition noted that when people engage in effortful information processing, they often bolster beliefs, rationalise or justify their intuitions^36^. More recently, one account of misinformation consumption holds that people engage in 'identity-protective cognition'^33^, i.e. people maintain beliefs aligned with their political worldview as a way to express loyalty to their affinity group.

Moreover, cognitive reflection exacerbates biases in the evaluation of information, particularly about politics^26,30,32^. Individuals with higher cognitive reflection scores were more likely to engage in motivational biases^32,33^—although see Persson et al.^34^. A similar phenomenon was observed among individuals with greater science literacy and education^58^.

In the context of fake news detection tasks, several studies examined whether belief in headlines is associated with cognitive reflection and political concordance^16^. However, only a single study evaluated the hypothesis that partisan bias increases with cognitive reflection, but did not find evidence for such an effect^29^.

Further research is needed to test whether this amply reported phenomenon also applies to false statements detection.

Based on the above, we will evaluate whether cognitive reflection exacerbates the difference in overall belief between politically concordant and discordant statements.

*H3: Individuals' cognitive reflection score will have a positive association with the difference between overall belief for concordant and discordant statements (*Fig. 1C*).*

### RQ4: Is cognitive reflection associated with higher scepticism?

Consistent findings relate cognitive reflection and scepticism. For example, three studies found a negative correlation between the CRT score and religious beliefs^44,59–61^. Cognitive reflection is also positively associated with higher scepticism towards paranormal beliefs^60^, acceptance of scientific claims^62^, and rejection of conspiracy theories^63^.

If scepticism is more prevalent among individuals with a stronger propensity to engage in analytical thinking, we expect those individuals to be more cautious and rate statements as “false” more often—regardless of their veracity. In fact, a recent study reported a negative association between CRT scores and overall belief^29^.

We therefore predict that participants with higher CRT scores will be less likely to believe in political claims, regardless of their veracity.

*H4: Individuals' cognitive reflection score will have a negative association with overall belief (*Fig. 1D*).*

**Figure 2:**
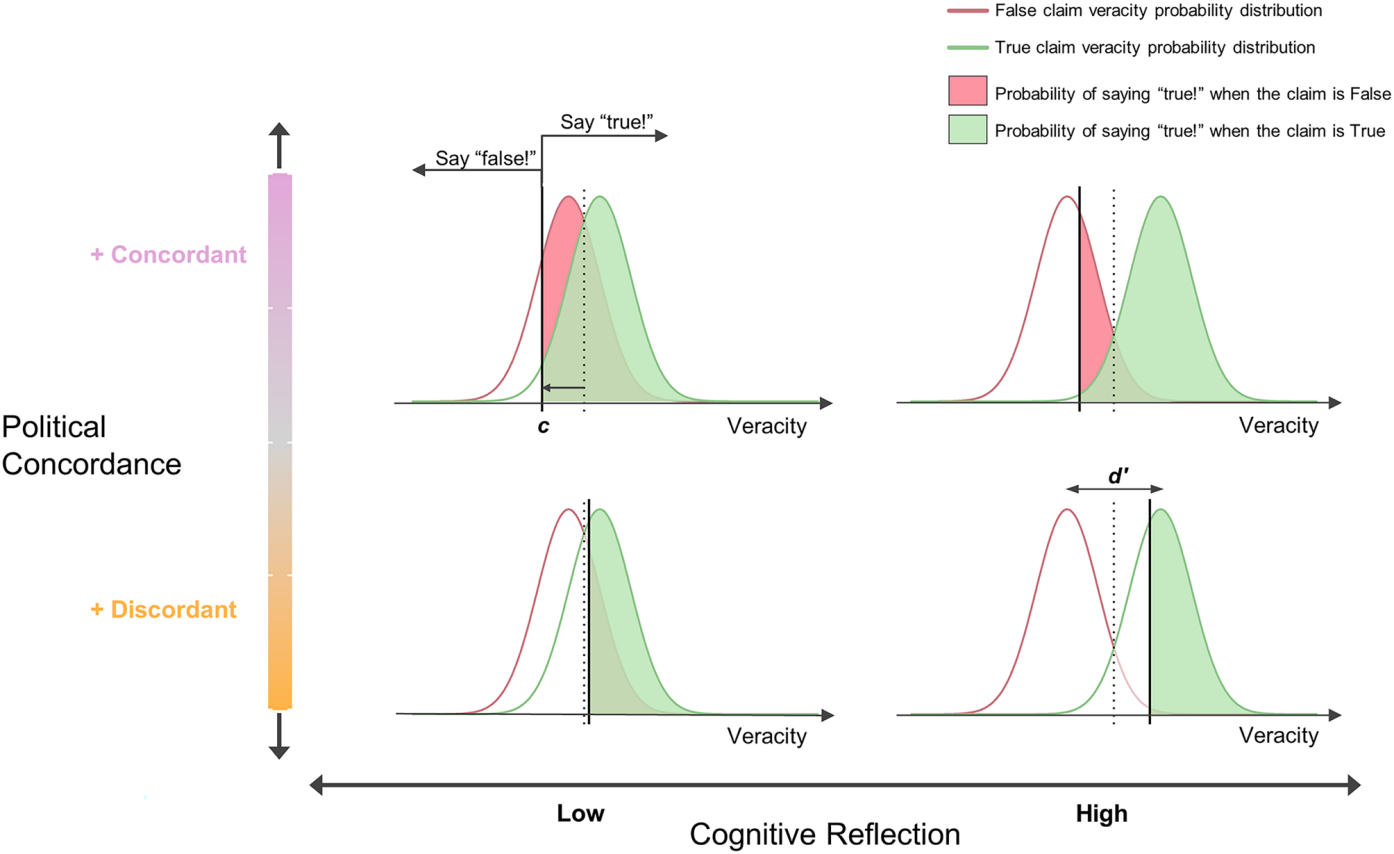
Joint representation of hypotheses under Signal Detection Theory. Signal Detection Theory (SDT) provides a unified framework to represent the decision process underlying the categorization of political statements as either true or false. The main assumption is that the presentation of a political statement to a participant triggers an internal decision signal that represents the veracity of that statement (inner-panels, horizontal axes). Then, if the statement's veracity is higher than a threshold (or criterion, c [black vertical line], also referred to as response bias), the participant will deem the statement “true”; otherwise, a “false” response will be produced. The criterion value is defined relative to the criterion of an ideal observer (dashed line), so that negative values imply a liberal criterion (resulting in more "true" responses) and positive values imply a conservative (or sceptical) criterion (resulting in more "false" responses). At the same time, this means that overall belief is represented by "-c”. An auxiliary assumption in this framework is that the veracity of true and false statements are normally distributed random variables (green and red lines, respectively), and that the mean of the “true statement” distribution is higher than the mean of the “false statement” distribution, both having the same variance. The difference between these means is d′ (truth discernment ability), which represents how "easy" it is to tell apart true from false statements (for undistinguishable statements, d′ is 0). Note that the ideal observer sets the criterion midway between the means of the true and false distributions when p(true) = p(false) = 0.5. To illustrate how our hypotheses (see Table 1 and Fig. 1) map onto this framework, we show four scenarios that derive from the crossing of the two variables of interest: individuals' Cognitive Reflection and statements' Political Concordance (outer x and y axes, respectively). H1 predicts that higher cognitive reflection will be associated with higher truth discernment ability (d′), regardless of the statements' political concordance. This is reflected in the spreading of the true and false distributions from low (left inner panels) to high (right inner panels) cognitive reflection. H2 predicts that overall belief (-c) will increase with political concordance, which is reflected in the shift of c (relative to the ideal observer, dashed line) to the left of the veracity axis from discordant (lower inner panels) to concordant (upper inner panels) statements. Importantly, in line with H3, this shift will be larger for higher cognitive reflection, i.e., the effect of political concordance (partisan bias) will be "amplified" by cognitive reflection. Finally, H4 predicts that higher cognitive reflection will be associated with higher scepticism (i.e., lower overall belief), which is reflected in the shift of c (relative to the ideal observer, dashed line) to the right of the veracity axis from low to high cognitive reflection. To facilitate understanding of how SDT parameters (d′ and c) interplay, we developed an interactive app offering relevant visualisations (https://bit.ly/SDT-app).

To test our hypotheses we built a model relying on SDT. To illustrate the basics of an SDT model we offer an interactive app (https://bit.ly/SDT-app). In turn, Fig. 2 shows the representation of our hypotheses in the context of the model.

Although the expected SDT indexes *d′* and *c* can be estimated for each participant and condition independently^29^, to test our hypotheses we will use a single multi-level SDT model to predict the binary response of each participant for each statement (True/False) and we will estimate parameters using Bayesian methods. One of the advantages of this approach compared to point estimates is that we avoid ad-hoc corrections when hit or false alarm rates are 0 or 1 (either by participant or by statement), a fairly common situation especially when the number of trials is low^64^. Moreover, our process model of the belief in misinformation goes beyond a mere mathematical description of behaviour and aims to understand how individuals make a decision based on the available information^65^.

Importantly, when viewed with the lens of SDT, partisan bias reflects the effect of political concordance on response bias, i.e. a shift in the propensity to believe a headline is true when it agrees or disagrees with the ideological view of the individual, regardless of his own truth discernment ability^20,29^. This is the key advantage of using SDT. An increase in truth discernment ability with cognitive reflection is fully compatible with an increase in partisan bias with cognitive reflection.

In sum, our study contributes to the growing literature aiming to understand belief in misinformation^16,27–29^ and will serve as a conceptual replication of previous studies such as Batailler et al.^29^.

## Methods

### Ethics information

The study has been approved by the Ethics Committee of “*Centro de Educación Médica e Investigaciones Clínicas*” (protocol ID 435) and was performed in line with the principles of the Declaration of Helsinki. This study was conducted online. Participants provided informed consent before the experiment and were debriefed at the conclusion of the study. No monetary compensation was awarded to participants.

### Experimental design

**Figure 3:**
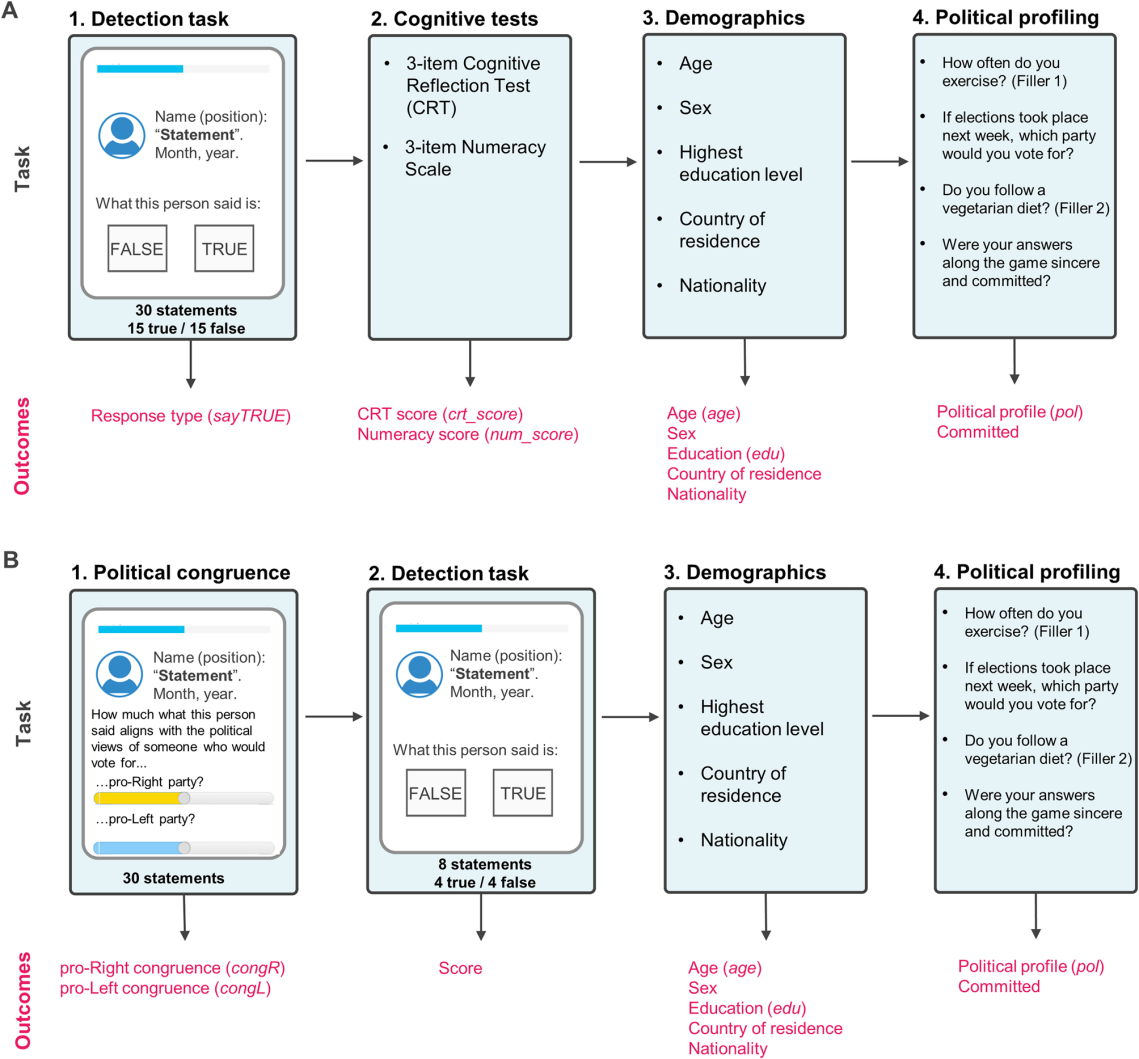
Experimental procedures. App-flow diagrams represent the sequential stages (tasks) of the two experimental procedures described in the “Experimental design” section. Below each stage-box, the corresponding outputs (description and variable names in italic type font) are detailed in magenta. These outputs were the data that were analyzed and/or used as exclusion criteria (see “Sampling plan” and “Data analysis” sections). (**A**) The main app comprised four stages: (1) Detection task, in which participants judged whether each of the 30 statements is false or true; (2) cognitive test; (3) demographic questions; and (4) political profiling (with two filler questions). (**B**) The calibration app encompassed four stages: (1) Participants rated (with sliders) the political congruence for each of the 30 statements, i.e. the degree of alignment between the statement and the political beliefs of a pro-Right (yellow) or pro-Left (light blue) individual; (2) a short version of the detection task from the main app; (3) and (4) were the same as the last two stages from the main app. A demo version of each app is accessible at https://bit.ly/main-app-demo and https://bit.ly/calibration-app-demo.

To test our hypotheses, we implemented an experimental procedure as a web app using R-Shiny^66^, a powerful framework for seamless cross-device implementation of online behavioural tasks^67^. This allowed us to collect large amounts of data online from participants using any type of device (e.g., phones, tablets, desktop computers). This main-app consists of 4 main stages (Fig. 3A):

1. Detection task: Participants completed a two-alternative forced choice (2-AFC) task in which they had to decide whether a political statement was true or false. These were 30 contemporary statements (15 true, 15 false) made by political leaders and elected officials in Argentina, selected after approval of the pre-registered protocol (Table S1). The veracity or falsehood of these statements was determined a priori by an independent local fact-checking organisation, Chequeado (http://www.chequeado.com), the only fact-checking agency in Argentina affiliated with the International Fact-Checking Network (https://ifcncodeofprinciples.poynter.org/). We did not deceive or mislead participants. All statements were statements actually said by the given politician. All statements shared the same structure (see Fig. 4 for a real example):
  *Political leader/elected official’s name (position):*
  ***“Statement”.***
  *Month, year.*
  Note that stimuli will not include any reference to a media outlet. Having this information may lead participants to use the perceived credibility of the media outlet as a cue to decide if a statement is true or false^40,41^. Then, it will make it difficult (if not impossible) to distinguish the effect of the trustworthiness of the media outlet with the effect of congruence with the statement itself. All participants will judge the same 30 statements (i.e., within-subjects design), but the order of presentation of the statements will be randomised for each participant.

**Figure 4:**
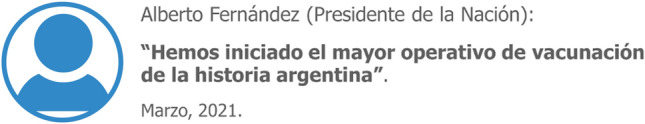
Example of stimulus. Each stimulus had the same structure (see “Experimental design”): A header with the name of the political leader and their current political position; the statement in bold type font and in between quotation marks; month and year when the statement was issued. In the actual experiments, instead of an image placeholder, a picture of the political leader (their current Twitter profile picture or, if not available, their official website’s profile picture) was displayed to the left of the statement. As an example, we show a statement from the President of Argentina, Alberto Fernandez. For the full list of stimuli, see Table S1.
2. Cognitive tests: participants completed a 3-item CRT^42^ and a 3-item Numeracy Test^49^. We wish to explicitly acknowledge that our original plan, as outlined in our registered Stage-1 protocol, involved using a 6-item CRT^68^ to gain a more nuanced understanding of participants' cognitive reflection. Regrettably, during the data collection process, an oversight occurred, and we inadvertently used the 3-item version of the CRT instead. We understand the significance of adhering to registered protocols to ensure the validity and transparency of our research. Although this constitutes a limitation of our study, we firmly believe that this unintentional deviation did not have a meaningful impact on the core research questions, data analysis, or conclusions drawn from our study. Instructions emphasized to not take too long nor Google-search the answer. Each item appeared on a separate screen, following the same order for all participants.
3. Demographic questions: We asked participants’ age, sex, highest education level, country of residence, and nationality. All questions were displayed in that order simultaneously on the same screen.
4. Political profiling: Argentina is a politically-polarized country^52^ with two major coalitions: "Juntos por el Cambio" (center-right wing) and "Frente de Todos" (center-left wing). To determine the political affiliation of participants, we inquired about their voting preferences in a hypothetical presidential election scheduled for the week following the experiment. For the sake of clarity, we will refer to voters of "Juntos por el Cambio" as pro-Right and voters of "Frente de Todos" as pro-Left. We would like to clarify that this paragraph aims to address a specific inconsistency in the Stage 1 registered report protocol. While the figure detailing the experimental procedures correctly indicated the question as "which party would you vote for?", an inadvertent reference to the question as "which candidate would you vote for?" was present in the text of the Stage 1 approved protocol. This discrepancy prompted us to rectify the text in the Stage 2 protocol to ensure alignment with the information presented to participants and correctly depicted in Fig. 3 (Fig. 5 in the Stage 1 approved protocol).

**Figure 5:**
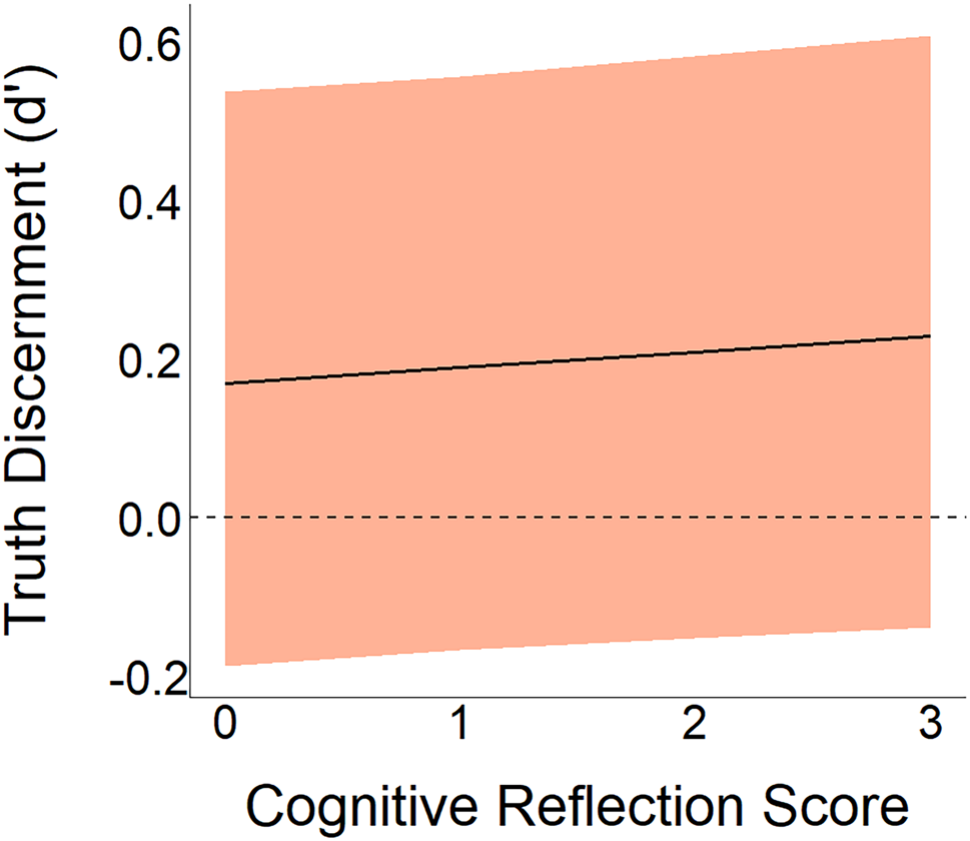
Relationship between CRT Score and Truth Discernment (H1). Our model suggests a positive link between analytical thinking propensity and truth discernment ability (${\delta }_{crt\_score}$ in Eq. 1a), though not reaching the registered threshold for reliability (see Table 3 for more details). The solid line represents the expected posterior median, while the shaded region depicts a credibility region corresponding to the 95% quantile interval of the posterior distribution.
5. This question was in-between two filler questions about physical activity frequency and whether they follow a vegetarian diet. Additionally, we explicitly asked whether participants were sincere and committed to the instructions (i.e., task engagement) while responding during the procedure.

We briefed instructions, set a temporal deadline for each trial of the detection task, and included a progress bar along the experimental procedure. The approved registered temporal deadline was set to 10 s. However, during the selection of fact-checked contemporary statements, we realized that the statement lengths were longer than anticipated. Considering the average reading speed in Spanish, we extended the deadline to 20 s to prevent data loss resulting from timed-out responses. Finally, after saving their data to our server, participants were informed about their performance (number of correct responses), and the actual veracity of all 30 statements were disclosed, followed by a request to not share this information.

To achieve our aim of understanding the effect of political concordance on overall belief we needed to measure the congruence between each statement and the political views of both a pro-Right and a pro-Left individual. Although statements were pre-selected to—a priori—span low and high congruence with either political profile, to determine the precise congruence of each statement we ran a political-statement calibration experiment. This calibration experiment—implemented as an app—was completed by an independent participant sample and consisted of four stages (Fig. 3B):

1. Political congruence: Participants rated how much they believed each of the 30 political statements we used for the detection task in the main app align with the political views of someone who would vote for either "Juntos por el Cambio" (center-right wing coalition) or "Frente de Todos" (center-left wing coalition) using two sliders (one for each political wing) ranging from “nothing” to “a lot”. All participants rated the same 30 statements (i.e., within-subjects design), but the order of presentation of the statements was randomised for each participant.
2. Detection task: Participants completed a reduced version of the main-app detection task with a fixed subset of 8 statements (4 true, 4 false) and received feedback on their performance (score). The order of presentation of the statements was the same for all participants.
3. Same as step 3 in the main app.
4. Same as step 4 in the main app.

Finally, once data was saved on our server, participants learned their score and the actual veracity of all statements—with a request to not share this information. Code and a running version of each app are accessible from the project’s OSF repository (https://osf.io/mhsr8/).

Importantly, as online data collection comes at the cost of uncontrolled conditions in the collection process (e.g., distractions coming from the participants’ surroundings), we performed data quality checks that aim to reduce the impact of these conditions. To this end, we recorded response times for each trial of the detection task and the cognitive tests in the main app, and for each trial of the political valence stage of the calibration app. This information allowed us to identify and remove all trials that were faster than a perceptual-latency threshold, and slower than a reasonable deadline for the cognitive tests (see “Data analysis” for details), as these data points are likely a by-product of uncommitted impulsive responding (or unintentional clicks/screen-taps) and distraction (or Google-searching the answers), respectively.

### Sampling plan

The sample size for the main study was determined to guarantee that the Bayes Factor (BF) is at least 10 times in favour of each experimental hypothesis over the null hypothesis (i.e., null effect).

To perform the BF analysis, we first generated data (for details, see Supplementary Information) under our experimental hypotheses by:

- setting the parameter values representing H1, H2, and H4 (see Table 1) at the effect sizes reported in a previous review studying belief in misinformation^16^.
- setting the parameter value representing H3 at a conservative “small” effect size^69^.

Then, we fitted our model (see “Data Analysis”, Eq. 1) and computed the BF using the Savage-Dickey density ratio method^70^ for each of the four experimental hypotheses over a null hypothesis (i.e. null effect). We repeated this process for different sample sizes until the BF for each hypothesis was at least 10 times in favor of the experimental over the null. We found that this was achieved with N = 1200 (${\delta }_{crt\_score}$: BF_10_ > 100; ${\lambda }_{pol\_concord}$: BF_10_ > 100; ${\lambda }_{pol\_concord:crt\_score}$: BF_10_ = 33; ${\lambda }_{crt\_score}$: BF_10_ > 100). The planned sample size for the calibration app was also 1200, as there are no hypotheses related to the data we obtained with this app.

### Participants

Participants accessed the main app and the calibration app via shortened URL links, which redirected them to the Shiny server hosting the apps (running in a Donweb [donweb.com] cloud server with 2 GB of memory).

We recruited N = 1353 participants for the main study (mean age 57.9, SD_age_ = 12.2; 669 women, 675 men, 9 other; 722 pro-Left, 631 pro-Right) and N = 1210 for the calibration task (mean age 58, SD_age_ = 12.4; 603 women, 613 men, 1 other; 531 pro-Left, 414 pro-Right, 272 other). See demographic details in Figs. S1 and S1. Participants were recruited using multiple approaches. The apps’ links circulated on the authors’ corresponding Universities’ email distribution lists of volunteers. It was also shared over social media through the authors’ lab accounts. To stimulate the participation of politically-polarized individuals, we also shared an invitation to participate in the study on the comment sections of the main news websites. Upon connection to our server, instructions were displayed and informed consent was prompted. All subjects had to give informed consent, which required them to confirm they were at least 18 years old in order to participate. All data collected from the main app was saved on our server but was only included in our analysis if participants met the following criteria:

- were Argentinian (determined by the nationality question in the demographics stage of the app).
- answered “yes” to the question about sincerity and commitment.
- were polarised (i.e., those who answered either “Frente de Todos” and “Juntos por el Cambio” on the political profiling question).
- responded in more than 750 ms and less than 10 s in—at least—75% of the detection task trials.
- responded in more than 1 s and less than 60 s to—at least—two of the three cognitive reflection items.
- responded in more than 1 s and less than 60 s to—at least—two of the three numeracy scale items.

We acknowledge that the last two criteria represent a deviation from the cutoff specified in the approved Stage 1 protocol, i.e. 75% of the items responded to within the specified time. We recognize that using percentages to express these criteria may not have been the most appropriate approach. Considering that both the numeracy scale and the CRT consist of only three items each, we have now opted for a more explicit expression of these criteria.

Similarly, all data from the calibration app was saved on our server but was only be included in our analyses if participants met the following criteria:

- were Argentinian (determined by the nationality question in the demographics stage of the app).
- answered “yes” to the question about sincerity and commitment.
- responded in more than 750 ms in—at least—75% of the political valence stage trials.

We tracked the number of participants that met the inclusion criteria and stopped data collection when reaching the pre-registered goal of 1200 participants on each app.

We collected data for both apps online and serially: First for the main app and then for the calibration app. As pre-registered, both data collection and analysis were blind. Further, participants were anonymous. Data obtained from the calibration app allowed us to estimate the average difference between pro-Right and pro-Left congruence of each statement, termed political valence (see “Data Analysis” and Table 2). Data obtained from the main app allowed us to estimate the association of political concordance and cognitive reflection with overall belief and truth discernment (see, Eqs. 1a and 1b below), while controlling for age, highest education level, and numeracy score.

**Table 2:** Glossary.

Variable name	Applies to	Meaning	Value range	Referred to as
Belief	Individuals	The probability of saying true, regardless of the veracity of the statement	Continuous min: 0 max: 1	π
Truth discernment	Individuals	The ability to tell apart true from false statements	Continuous	*d′*
Overall belief	Individuals	The propensity to say true regardless of the veracity of the statement	Continuous	*-c*
Scepticism (or response bias)	Individuals	The propensity to say false regardless of the veracity of the statement	Continuous	*c*
CRT score	Individuals	The score obtained by an individual in the 3-item Cognitive Reflection Test, defined as the number of correct responses	Continuous	*crt_score (standardised version of the CRT score)*
Numeracy score	Individuals	The number of correct responses obtained by an individual in the 3-item Numeracy Test^49^	Continuous	*num_score (standardised version of the numeracy score)*
pro-Right congruence	Statements	An estimate of how likely a pro-Right individual would agree with a statement	Continuous Min: 0 Max: 1	*congR*
pro-Left congruence	Statements	An estimate of how likely a pro-Left individual would agree with a statement	Continuous Min: 0 Max: 1	*congL*
Political valence	Statements	The difference between pro-Right and pro-Left congruence	Continuous Min: − 1 (pro-Left) Max: 1 (pro-Right)	*delta_cong*
Political profile	Individuals	Voting preference	Categorial Levels: pro-Right, pro-Left, other	*pol*
Political concordance	Statements and individuals	Alignment between statement’s political valence and individual’s political profile It is computed as the political valence with sign flipped for pro-Left individuals	Continuous	*pol_concord (standardised version of political concordance)*
Partisan bias	Individuals	The effect of political concordance on overall belief	Continuous	− λ_*pol_concord*_

The pro-Right and pro-Left congruence estimates will be inferred from data collected with the calibration app (see “Data Analysis” sections).

### Data analysis

To test our hypotheses (see Table 1), we implemented a hierarchical equal-variance SDT model^64,71–74^ that represents the statement-veracity judgement as a Bernoulli process (Fig. 2):

$$sayTRU{E}_{i} \sim Bernoulli({\pi }_{i})$$

$${\pi }_{i}=\Phi ({d}_{i}{\prime}*isTRU{E}_{i} - {c}_{i})$$

where—for trial *i*—*sayTRUE* is any given participants’ binary response (either 1: true, or 0: false) to the detection task. This response is modelled as a realisation of a Bernoulli trial with probability π. This probability results from mapping z-scores to probability with the normal cumulative density function ($\Phi$), which bounds values between 0 and 1. These z-scores are determined by the truth discernment parameter (*d′*), and response bias (c) (the opposite of overall belief, see Glossary). Last, *isTRUE* is a vector of ground truth of the statements (1: true, 0: false, determined a priori by Chequeado), which “turns on” the increase in the probability (in z-score) of saying true when a statement is true.

Importantly, to understand how political concordance and cognitive reflection relate to each of the SDT parameters, we built a hierarchical linear model for *d′* and *c*. As more education and younger age are associated with greater scores in the CRT^75^, we included participants’ age and education level in our model to account for these associations. Specifically, the predictors included are:

*pol_concord:* a numerical variable (standardised) indicating the political concordance for a given statement and participant. This variable was obtained from the analysis of the calibration app data (see below).
*crt_score*: a numerical variable (standardised) indicating the score—defined as the number of correct responses—in the cognitive reflection test for each participant, with positive values meaning higher than the mean scores, and negative values, lower than the mean.
*num_score*: a numerical variable (standardised) indicating the score—defined as the number of correct responses—in the numeracy scale test for each participant, with positive values meaning higher than the mean scores, and negative values, lower than the mean.
*edu*: a categorical variable indicating the participants’ highest education level. These categories are indexed with sub-index* m* (m = 1,2,3) and we will set “*high-school complete*” as the reference category.
*age*: a numerical variable (standardised) indicating the participants’ age.

Next, we report the specification for the linear predictors of *d′* and *c*.

1a $$\begin{aligned} {d{\prime}}_{i} & = {\delta }_{Intercept}+{\delta }_{Intercept, i{d}_{i}}+{\delta }_{Intercept, statemen{t}_{i}}+{\delta }_{crt\_score}\times crt\_scor{e}_{i} \\ & \quad +{\delta }_{pol\_concord} \times pol\_concor{d}_{i} +{\delta }_{pol\_concord:crt\_score} \times pol\_concor{d}_{i} \times crt\_scor{e}_{i} +{\delta }_{num\_score} \times num\_scor{e}_{i} \\ & \quad +{\delta }_{age } \times ag{e}_{i} + {\delta }_{edu, m  } \times  ed{u}_{i,  m} \end{aligned}$$

1b $$\begin{aligned} {c}_{i} & = {{\lambda }_{Intercept}+{\lambda }_{Intercept, i{d}_{i}}}+{{\lambda }_{Intercept, statemen{t}_{i}}}+{\lambda }_{crt\_score} \times crt\_scor{e}_{i}+{\lambda }_{pol\_concord} \times pol\_concor{d}_{i} \\ & \quad +{\lambda }_{pol\_concord:crt\_score} \times pol\_concor{d}_{i} \times crt\_scor{e}_{i}+{\lambda }_{num\_score} \times num\_scor{e}_{i} \\ & \quad+{\lambda }_{age } \times ag{e}_{i} + {\lambda }_{edu, m } \times ed{u}_{i, m} \end{aligned}$$

We accounted for by-participant and by-statement variability by allowing ${\delta }_{Intercept}$ and ${\lambda }_{Intercept}$ to vary by participant (${\delta }_{Intercept, i{d}_{i}}, {\lambda }_{Intercept, i{d}_{i}}$) and statement (${\delta }_{Intercept, statemen{t}_{i}}, {\lambda }_{Intercept, statemen{t}_{i}}$), respectively. We estimated model parameters with Bayesian inference methods using the R package brms^76^.

After estimating the model, we were able to answer our research questions directly from the model output. Specifically, the coefficients of interest (see Table 1 and Fig. 1) were:

- ${\delta }_{crt\_score}$ to answer RQ1
- ${\lambda }_{pol\_concord}$ to answer RQ2
- ${\lambda }_{pol\_concord:crt\_score}$ to answer RQ3
- ${\lambda }_{crt\_score}$ to answer RQ4

We obtained 8000 samples of the posterior distribution for each coefficient from the model output, which we then used for hypothesis testing. Namely, since all of our hypotheses are directional, we tested them by computing the proportion of posterior values that are below 0 for ${\lambda }_{pol\_concord}$ and ${\lambda }_{pol\_concord:crt\_score}$, and above 0 for ${\delta }_{crt\_score}$ and ${\lambda }_{crt\_score}$. If the proportion was greater than 0.95, then we interpreted that there was reliable evidence supporting the corresponding hypothesis. If the proportion was not greater than 0.95, then we interpreted that there was not reliable evidence supporting the corresponding hypothesis.

Next, we describe the analysis we performed with data from the calibration app. The purpose of the calibration app was to obtain an average rating of the pro-L and pro-R political congruence for each of the 30 political statements (coined *congL* and *congR*, respectively*)*. These ratings were collected using two continuous sliders in the app (see Design, Fig. 3B).

We jointly analysed these ratings with a multivariate zero-one-inflated-beta (ZOIB)^77^ Generalised Linear Mixed Model (GLMM) using Bayesian methods with the R package brms^76^. This mixture-of-distributions modelling approach is a better representation of the putative underlying generative process of subjective ratings (bounded between 0 and 1) which allows to dissect discrete events (zeros and ones, i.e., extreme responses) from continuous ratings using four distributional parameters: Mean (μ) and precision ($\phi$) of the reparameterized beta distribution, probability of binary rating (α), and probability that a rating is 1, given a binary rating (γ). In equation form:

2a $$congL\sim (1 -Bernoulli(\alpha )) \times Beta(\phi \times \mu ,\phi \times(1-\mu )) + Bernoulli(\alpha )\times Bernoulli(\gamma )$$

2b $$congR\sim (1 -Bernoulli(\alpha )) \times Beta(\phi \times \mu ,\phi \times (1-\mu )) + Bernoulli(\alpha ) \times Bernoulli(\gamma )$$

Note, *congL* (pro-Left congruence) and *congR* (pro-Right congruence) are the dependent variables representing the degree of perceived congruence between a statement and the political views of someone who would either vote for the center-right coalition or the center-left coalition, respectively.

Crucially, our approach allowed us to specify a linear model on each of the four parameters of the probability distribution mixture. Most relevant to our analysis, we built a linear model for the parameter μ in Eqs. (2a and 2b) to estimate the mean rating for each political statement (i.e. for each dependent variable), while accounting for by-participant variability. Specifically, the predictors included were:

*statement*: a categorical variable indicating the statement ID. These categories were indexed with sub-index* j* (j = 1,…,29) and the first statement (ID = 1) was set as the reference category.
*pol:*: a categorical variable indicating the participants’ political profile (i.e., whether they answered “Frente de Todos” (center-left coalition), “Juntos por el Cambio” (center-right coalition) or “other” to the political profiling question). These categories were indexed with sub-index* k* (k = 1,2) and we set “*other*” as the reference category.
*edu*: a categorical variable indicating the participants’ highest education level. These categories were indexed with sub-index* m* (m = 1,2,3) and we set “*high-school complete*” as the reference category.
*age:* a numerical variable (standardised) indicating the participants’ age.

Next, we report the specification for the linear predictor of μ (the other distributional parameters -$\phi$, α, and γ- are modelled with an overall intercept with by-subject variability). Note that we used a logit link function to bound the values in the response scale and to aid model convergence.

3 $$logit({\mu }_{D{V}_{i}}) ={\beta }_{Intercept, DV, i{d}_{i}}+{\beta }_{statement, DV, j} \times statemen{t}_{i, DV, j}+{\beta }_{pol, DV, k } \times po{l}_{i, DV, k}+{\beta }_{age, DV } \times ag{e}_{i, DV} + {\beta }_{edu, DV, m } \times ed{u}_{i, DV, m}$$

Note, *DV:* dependent variable (either *congL* or *congR*), *id*: participant ID; *i*: trial index;* j*: statement index, *k:* political profile index, *m:* education level index.

To estimate the model, we specified weakly-informative—yet regularising—priors that provide sensible a priori data distributions. After estimating the model, we computed the expected population values (i.e., for an average participant) of each dependent variable for each statement, across political profile and education level index, and for mean age. Last, we obtained the political valence of each statement by subtracting the expected *congL* from the expected *congR* values. This way, positive values for political valence corresponded to political statements that are more aligned with right-wing party voters, and negative values represented higher alignment with left-wing voters. The results from this analysis (Fig. S3) were saved as a 30-by-2 dataframe (one row per political statement, one column with the statement ID, and one with political valence values) that we used for the main analysis. Last, political concordance (i.e., alignment between political valence of the statements and political profile of the participants) was computed as the political valence for pro-Right participants and as – 1 × political valence for pro-Left participants. This way, positive values for political concordance represented belief-congruent political statements, and negative values, belief-incongruent political statements.

We applied a response-time based filter by which we removed trials with response times faster than 750 ms in the political congruence stage of the calibration app, as well as in the detection task of the main app. Items with response times faster than 1 s or slower than 60 s in the cognitive reflection test or the numeracy scale were also discarded from further analysis.

Given that data analytic choices may influence results^78,79^, we also report the results of our analysis without including age and education level as predictors in our models, Eqs. (1a and 1b) (Table 4).

Finally, we included in the project’s OSF repository (https://osf.io/mhsr8/) R scripts that implement the described workflow and feasibility of our analysis plan (see “Parameter recovery (pilot data)” and Supplementary Information) for both the main and the calibration apps.

### Parameter recovery (pilot data)

To demonstrate the feasibility of our methodological approach, we performed a parameter recovery analysis^80^ using fake data that we generated (with fixed known “true” parameter values) following the structure of the real data we collected with the calibration and main applications (apps)—see “Data analysis”.

This analysis intended to assess whether using our model we could confidently recover the known parameters values from fake data generated with said true values. If the recovery was successful, the modelling strategy was proven reliable. Our results showed that the recovery was successful (Fig. S4). We describe in full the parameter recovery analysis and results in the Supplementary Information with the corresponding reproducible code and simulated data included in the project’s OSF repository (https://osf.io/mhsr8/).

## Results

We recruited a sample of 1353 politically polarized adults in Argentina. Participants were shown 30 statements (half true, half false) made by Argentinian politicians that had already been classified by the fact-checking agency Chequeado. After seeing each statement, participants were asked “What this person said is true or false?”. In a calibration test, an independent pool of participants rated the pro-Left and pro-Right alignment of each statement. Participants also completed a cognitive reflection test^42^. For more details, see Methods.

We used SDT to model the decision process and to link each of our hypotheses to a single model parameter. The outcome variable (participants’ response: True/False) depends on truth discernment (d′), the SDT parameter that captures the participant’s ability to correctly discriminate between true and false statements, and response bias (c), the SDT parameter that represents the tendency to classify the statement as true or false. For clarity purposes, instead of reporting results in terms of c, we use “overall belief” (-c), a terminology introduced by Pennycook and Rand^16^ that refers to the tendency to accept a statement as true (see the accompanying SDT app for details https://bit.ly/SDT-app and Table 2 for a Glossary).

We first report the results of the approved registered analysis and then move to an exploratory analysis. In all cases, since we used Bayesian inference methods to fit the model, we obtained posterior samples for each coefficient and report the median and a 95% quantile interval (CI95). As registered, we tested our hypothesis by counting the proportion of posterior samples above or below zero (depending on the hypothesis, as shown in Table 1) and comparing this value to a reliability threshold of 95% (refer to Methods for further details).

### Registered analysis

A summary of the four estimated coefficients corresponding to each of the four hypotheses is shown in Table 3.

**Table 3:** Estimated coefficients.

Coefficient (Θ)	Median	CI95	Hypothesis	P(Θ > 0)	P(Θ < 0)
${\delta }_{crt\_score}$	0.016	[− 0.015, 0.046]	H1	0.85	0.15
${\lambda }_{pol\_concord}$	− 0.663	[− 0.685, − 0.640]	H2	≅ 0	≅ 1
${\lambda }_{pol\_concord:crt\_score}$	− 0.016	[− 0.037, 0.006]	H3	0.08	0.92
${\lambda }_{crt\_score}$	0.039	[0.006, 0.072]	H4	0.99	0.01

Median, 95% credibility interval and hypothesis test.

#### Is cognitive reflection linked to improved discernment? (RQ1)

To answer this question, linked to our hypothesis H1, we focus on whether the score in the cognitive reflection test was associated with truth discernment ability, i.e. coefficient ${\delta }_{crt\_score}$ in Eq. (1a). We analysed the posterior samples and found a positive point estimate for ${\delta }_{crt\_score}$ (median = 0.016, CI95 = [-0.015, 0.046]) (Fig. 5), but only 85% of the samples were positive (p(${\delta }_{crt\_score}$> 0) = 0.85). According to our pre-established criterion, where a proportion above 95% would be considered reliable evidence, we conclude that there is not reliable evidence for an association between cognitive reflection and truth discernment, casting doubts on our Hypothesis 1.

#### Does partisanship bias judgments? (RQ2)

**Figure 6:**
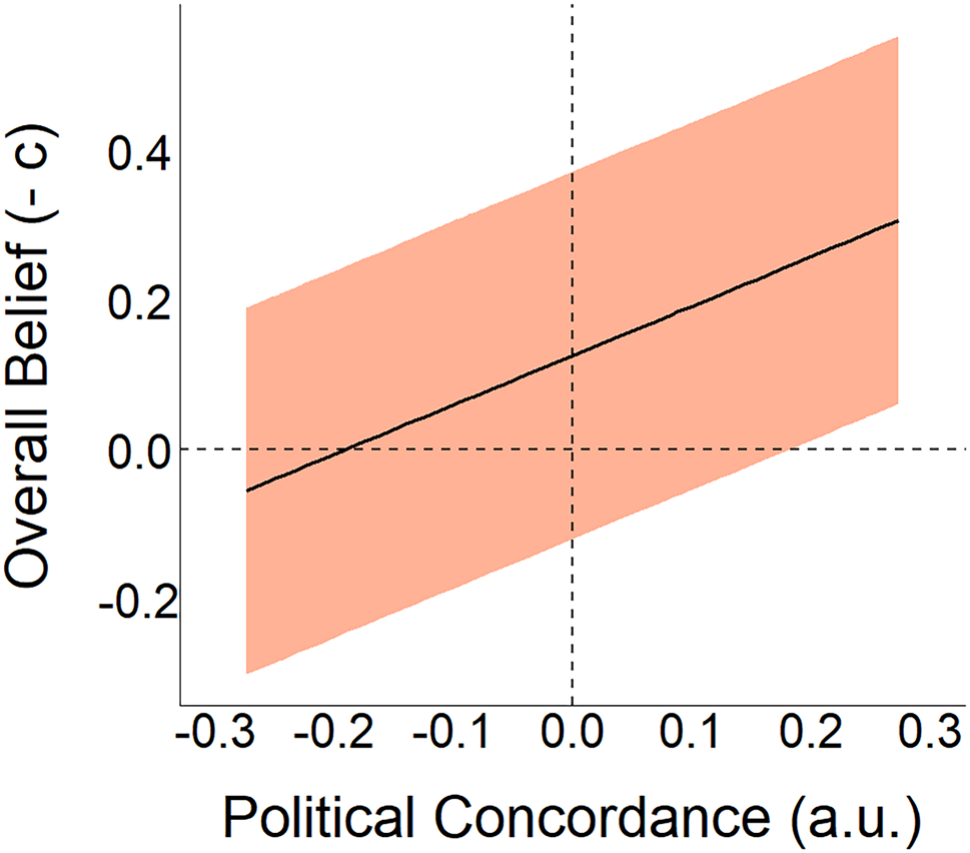
Partisanship is associated with shifted judgements about the veracity of political statements, i.e. partisan bias (H2). Political concordance of statements reliably predicts overall belief. For clarity, instead of plotting the expected relationship between response bias (*c*) and political concordance, we plot overall belief (-*c*) adopting the terminology introduced by Pennycook and Rand^16^. Thus, our data and model show that there is a reliable and positive association between political concordance and overall belief ($-{\lambda }_{pol\_concord}$, see Table 3 for more details). The solid line represents the expected posterior median, while the shaded region depicts a credibility region corresponding to the 95% quantile interval of the posterior distribution.

We hypothesised that overall belief in political statements was associated with political concordance (H2), i.e. the alignment between a statement's political valence and an individual's political profile. To test this hypothesis, we analysed the posterior distribution of the ${\lambda }_{pol\_concord}$ coefficient in Eq. (1b). We found a reliable (p(${\lambda }_{pol\_concord}$ < 0) ≅ 1) negative correlation (median = − 0.663, CI95 = [− 0.685, − 0.640]) (Fig. 6). Therefore, confirming our Hypothesis 2, we conclude that higher political concordance is associated with greater belief in that a statement is true, regardless of the actual veracity of that statement.

#### Does cognitive reflection amplify partisan biases? (RQ3)

**Figure 7:**
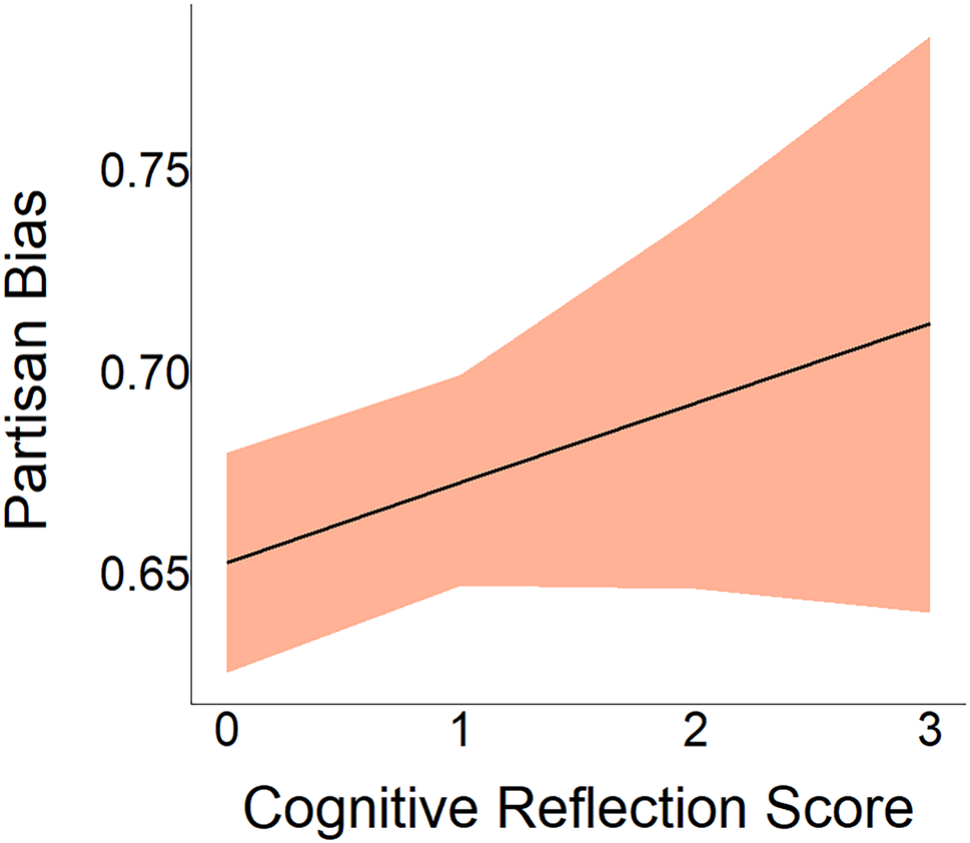
Association between cognitive reflection and partisan bias (H3). The impact of political concordance on overall belief seems to be enhanced in individuals with higher Cognitive Reflection Test (CRT) scores, yet the evidence falls short of the pre-established 95% reliability threshold ($-{\lambda }_{pol\_concord:crt\_score}$, see Table 3 for more details). The solid line represents the expected posterior median, while the shaded region depicts a credibility region corresponding to the 95% quantile interval of the posterior distribution.

We next investigate the correlation between cognitive reflection and partisan bias, defined as the effect of political concordance and overall belief (H3). To this end, we examined the posterior distribution of ${\lambda }_{pol\_concord:crt\_score}$ in Eq. (1b), the coefficient corresponding to the interaction between political concordance and the CRT score of an individual. We found a negative correlation (median = − 0.016, CI95 = [− 0.037, 0.006]) (Fig. 7), suggesting that the effect of political concordance on overall belief might be larger for participants with higher cognitive reflection scores. However, the posterior distribution revealed that only 92% of its mass lies below zero (p(${\lambda }_{pol\_concord:crt\_score}$ < 0) = 0.92). The effect does not meet the predefined threshold for reliability. Therefore, as registered, we conclude that there is no reliable evidence for an association between cognitive reflection and the ability to rationalize ideologically concordant information while dismissing discordant information.

#### Is cognitive reflection associated with higher scepticism? (RQ4)

**Figure 8:**
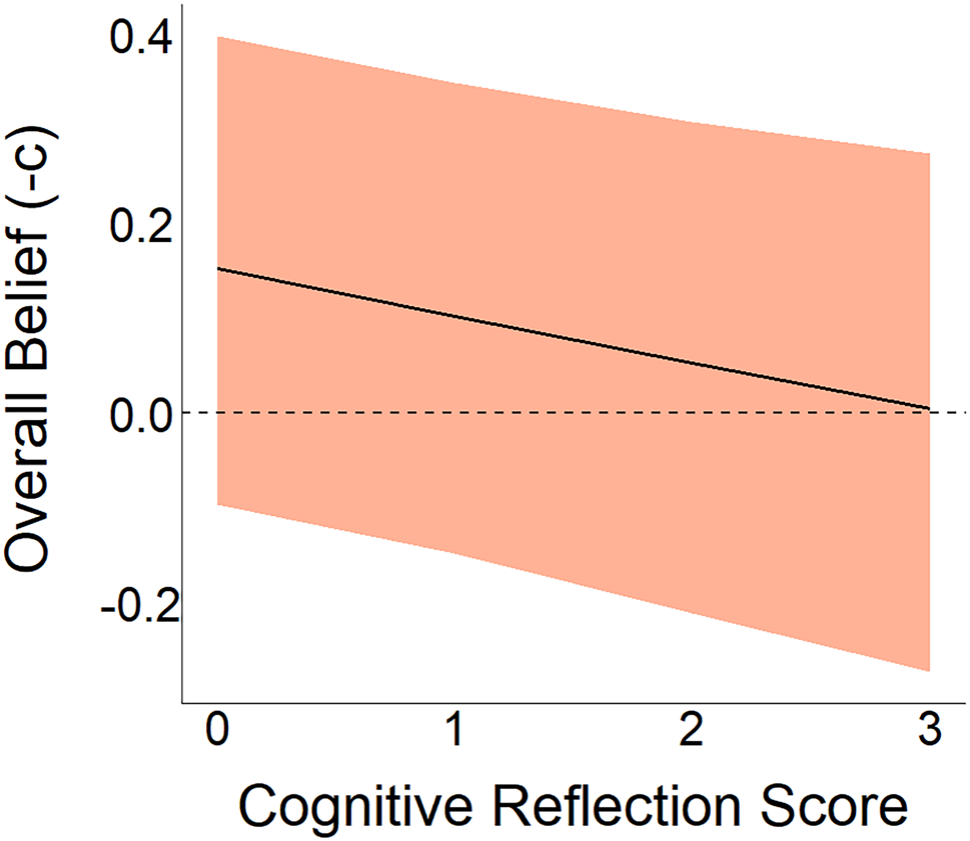
Cognitive reflection is associated with higher scepticism (H4). Our findings indicate that individuals with higher CRT scores exhibit a reduced inclination to accept statements at face value (${-\lambda }_{crt\_score}$, see Table 3 for more details). The solid line represents the expected posterior median, while the shaded region depicts a credibility region corresponding to the 95% quantile interval of the posterior distribution.

To assess the correlation between cognitive reflection and scepticism (H4), we analysed the posterior distribution of ${\lambda }_{crt\_score}$ in Eq. (1b), the coefficient of the linear relationship between response bias and the score in the cognitive reflection test. We found that there is a reliable (p(${\lambda }_{crt\_score}$> 0) = 0.99) positive correlation (median = 0.039, CI95 = [0.006, 0.072]). In other words, individuals with higher CRT scores had lower overall belief, regardless of their veracity (Fig. 8). Therefore, we conclude that higher cognitive reflection is associated with greater scepticism.

#### Replication without control variables

As registered, given that there data analytic choices may influence results^78,79^, we also we performed the same analysis detailed in Methods but removed the control variables *age* and *edu* in the linear model for *d′* and *c* (Eq. 1a and 1b), which is part of the SDT model specification. The results are detailed in Table 4. Overall, estimates with and without control variables closely match. Thus, the interpretation of our results holds.

**Table 4:** Estimated coefficients for the SDT model without control variables (age and edu).

Coefficient (Θ)	Median	CI95	Hypothesis	P(Θ > 0)	P(Θ < 0)
${\delta }_{crt\_score}$	0.020	[− 0.011, 0.050]	H1	0.91	0.09
${\lambda }_{pol\_concord}$	− 0.662	[− 0.685, − 0.639]	H2	≅ 0	≅ 1
${\lambda }_{pol\_concord:crt\_score}$	− 0.015	[− 0.038, 0.007]	H3	0.09	0.91
${\lambda }_{crt\_score}$	0.047	[0.014, 0.080]	H4	≅ 1	≅ 0

Median, 95% credibility interval and hypothesis test.

### Exploratory analysis

#### Does partisanship improve truth discernment?

In recent years, there has been a debate on whether partisanship facilitates or hinders the detection of misinformation^16,20,21^. This debate stems from the observation that individuals tend to exhibit two seemingly opposed patterns: (a) greater belief in news that aligns with their political ideology, regardless of its veracity; (b) better truth discernment when evaluating concordant news compared to politically discordant news. The former is addressed by our hypothesis 2. While our hypotheses did not explicitly address the association between political concordance and truth discernment, we present our findings on this matter to contribute to the ongoing discourse.

The coefficient ${\delta }_{pol\_concord}$ in Eq. (1a) corresponds to the correlation between truth discernment and political concordance. We analysed the posterior samples of ${\delta }_{pol\_concord}$ and found that there was a reliable (p(${\delta }_{pol\_concord}$> 0) ≅ 1) positive correlation (median = 0.076, 95% CI [0.046, 0.106]). Therefore, we conclude that higher political concordance is associated with better truth discernment. Please refer to Fig. S5 for a full comparison of all model coefficients.

## Discussion and conclusion

In this registered report, we investigated how partisanship and cognitive reflection influence the response of participants when they judge whether a statement made by a politician is true or false. Using Signal Detection Theory, we studied the influence of both factors on the ability to tell apart true from false statements (truth discernment ability) and the propensity to say true regardless of the veracity of the statement (overall belief).

First, we found reliable evidence of partisan bias, i.e. the effect of political concordance on overall belief (H2). Participants were more likely to believe in political statements as these statements were more aligned with their ideological worldview. This is consistent with mounting evidence showing that partisanship is one of the drivers of misinformation belief^16,81,82^. More specifically, our study extends previous results focused on factual information^83^ and fake news^16,27,29,82,84^ to statements made by politicians, a less explored format.

Replicating prior work^16,85^, we found, in an exploratory analysis, that political concordance was also associated with an increased ability to tell apart true from false statements. However, this association was lower in magnitude than partisan bias. The effect size of political concordance on overall belief was ~ 0.66 while the improvement of truth discernment with political concordance of statements had an effect size of ~ 0.08. This difference in effect size conceptually replicates results from a combined analysis of several studies on belief in fake news^16^.

Taken together, our results support the view that political concordance enhances truth discernment, albeit accompanied by a shift in response bias. Given the ongoing discussion regarding the influence of partisanship on belief in misinformation^20,21,27,28,85^, it is important to interpret the dual role of partisanship cautiously, in light of Signal Detection Theory. Although an improvement in truth discernment ability is undeniably advantageous, it merely establishes the upper limit to the accuracy participants can attain. It is noteworthy that an increase in truth discernment, coupled with a shift in overall belief, may even lead to a decline in accuracy, measured as the percentage of correct responses (refer to our SDT app: https://bit.ly/SDT-app).

Second, higher cognitive reflection was associated with greater scepticism, i.e. those that scored higher in the CRT were also less likely to believe in political statements, regardless of the veracity (H4). This is in agreement with previous research on belief in fake news^16,29^.

Finally, we did not find reliable evidence in favour of the two remaining hypotheses. Cognitive reflection did not demonstrate an improvement in truth discernment, as we hypothesised (H1). This outcome contrasts with the consistent evidence reported in several studies that specifically examined this question in the context of belief in fake news^16,29,86,87^. The underlying reasons for this disparity remain uncertain and warrant further investigation. Nonetheless, it is noteworthy to mention that the evidence supporting this hypothesis reached 85% (see Table 3). Although falling short of the pre-established reliability threshold, it still presents a moderate level of strength and should not be disregarded outright.

On the other hand, cognitive reflection did not reliably increase partisan bias (H3). This finding aligns with previous studies conducted by Batailler et al.^29^ and Gawronski et al.^87^, both of which are prominent works investigating the impact of cognitive reflection on partisan bias, as measured by the difference between response bias for ideology-congruent and ideology-incongruent news headlines. However, the posterior distribution's 92% mass below zero suggests a tendency in favor of the hypothesized effect (see Table 3), although the evidence does not meet the 95% predetermined threshold for reliability. This suggests the need for further investigations to fully ascertain the importance of this finding, especially considering the evidence that in certain circumstances, partisan biases are more prominent among individuals with higher cognitive ability^26,30,32,33^.

We acknowledge several limitations of our study. Firstly, we did not experimentally manipulate the cognitive effort exerted by participants when judging the veracity of statements, as some previous studies have done. These methods include imposing response deadlines^86,87^ or providing instructions to carefully deliberate on the statements^85^. While our study design does not allow for drawing causal inferences, this was not the primary objective. Our focus was on examining participants' propensity to engage in analytical thinking, which is an inherent individual psychological characteristic.

Secondly, it is crucial to recognize that the task employed in our study does not replicate the real-world context in which individuals encounter and process political discourse. Our task represents a controlled condition that simplifies the complexity of real-life political contexts. Consequently, the evaluations made by participants in our study may not necessarily reflect their evaluations of political statements in more natural and ecologically valid settings.

Thirdly, our original intention was to utilise an extended version of the 3-item Cognitive Reflection Test (CRT) proposed by Frederick^42^, as outlined in the approved Stage 1 protocol. The extended version, introduced by Primi et al.^68^, consists of a total of six items, including the original three items and three additional problems. The rationale behind using this extended version was to address a limitation observed in the original test, specifically the difficulty of the problems, which led, in some samples, to a considerable proportion of participants receiving a test score of 0^42^.

However, we unintentionally implemented the 3-item CRT instead, deviating from our initially registered Stage 1 protocol. The 6-item CRT could have proven advantageous, particularly to distinguish among participants that obtained a score of 0 in the CRT (Fig. S1B). However, it is essential to note that the 3-item CRT remains a valid measure for assessing cognitive reflection and all research questions and data analyses were conducted consistently. Therefore, even though the outcomes may have differed with the 6-item CRT version, we assert that this deviation does not undermine the credibility of our study.

Another concern is that answering the CRT problems correctly requires not only overriding intuitions but also a certain degree of numerical ability^75^. To address this potential problem, we administered a test of numerical ability to participants^49^ and controlled for numeracy in our statistical models. Furthermore, after approval of our Stage 1 protocol, an alternative version of the CRT with less numeric elements has been proposed^88^. The latter serves as an interesting alternative, addressing numeracy confounds, and has already been used in Sultan et al.'s study on belief in misinformation^86^.

Furthermore, the interpretation of partisan bias observed in our study warrants careful consideration. We cannot determine whether such bias is driven by motivational factors^27,28,85^ or the influence of biased prior knowledge acquired through exposure to partisan sources^35,57^. Therefore, while partisan bias exists, reflected in the effect of political concordance on response bias, it may not necessarily be a deliberate action taken by individuals to defend their in-group, but rather an interplay of cognitive processes and information sources.

It is also worth noting that our study used a sample of polarized participants, as measured with their voting preferences. However, a pivotal aspect, unmeasured in the current study, is the level of political engagement. It is highly probable that within our sample, there is a spectrum of individuals with differing degrees of interest in political issues. This is an important consideration, as individuals who pay less attention to politics may assess political issues differently, potentially expressing less partisanship than their politically engaged counterparts^89^.

From a methodological standpoint, we placed particular emphasis on conducting a pre-test, as suggested by Pennycook et al.^90^. Through our calibration experiment, we obtained nuanced estimates of the political alignment of each statement, avoiding binary valence assignments based solely on the party affiliation of the politician making the statement. Furthermore, building upon the insights highlighted by Batailler et al.^29^, SDT proves to be an appropriate framework for modelling the decision process underlying belief in misinformation. Notably, SDT allows for the disentanglement of truth discernment from overall belief and provides a precise conceptualization of partisan bias.

Additionally, to delve deeper into the mechanisms of partisan information processing and the role of cognitive reflection, we adopted a hierarchical Bayesian modelling approach to SDT, as recently utilised by Sultan et al.^86^. This approach goes beyond mere mathematical descriptions of behaviour and aims to understand how individuals make decisions based on available information. For further insights, future investigations may find value in employing a drift diffusion model approach, such as the one proposed by Derreumaux et al.^91^ and Hause Lin et al.^92^. This approach could offer a more comprehensive understanding of the complex factors influencing belief formation and decision-making processes.

Finally, while the spread of misinformation poses a global challenge, there is a widespread consensus among experts in the field of misinformation that more data from outside the US is necessary^93^. In particular, Spanish-speaking countries, including Argentina, have been largely underrepresented in misinformation studies (with a few exceptions: Merpert et al.^94^, Porter and Wood^95^, Espina et al.^96^ and Arechar et al.^97^). One notable distinction is that misinformation in the Global South, unlike the case of the US or Europe, tends to proliferate through WhatsApp, the most popular messaging platform in this region, characterized by unique features^98^. Additionally, Spanish-speaking communities face an increased vulnerability to disinformation, as evidenced by the reduced effectiveness of social media platforms in preventing the viralization of false messages in Spanish compared to English^99^. Therefore, by focusing on Argentina, our study also makes a valuable contribution to the literature on misinformation.

In sum, our study contributes to the understanding of misinformation belief, extends prior investigations by focusing on political statements rather than fake news, conceptually replicate previous findings, and shed light on a complex phenomenon that impacts society as a whole.

## Supplementary Information

Supplementary Information.

## Data Availability

All data (fake and collected) and materials are stored in the project’s OSF repository (https://osf.io/mhsr8/) which was accessible for peer-review, and is now public. Specifically, we stored the following data (in .csv and .Rda format) to the repository: Final dataset collected with the main app. Final dataset collected with the calibration app. Output dataset of political statements’ political valence, obtained from the calibration app analysis. Fake dataset generated in the main app parameter recovery analysis. Fake dataset generated in the calibration app parameter recovery analysis. Output dataset of political statements’ political valence, obtained from the calibration app parameter recovery analysis. All datasets have a corresponding codebook (in .csv) explaining the data structure. The following materials are also stored in the repository: The 30 political statements used for both apps in .png format. A .txt document containing the 3-item Cognitive Reflection Test used in the main app. A .txt document containing the 3-item Numeracy Scale used in the main app. A .txt document containing the demographic questionnaire used in both apps. A .txt document containing the political profiling questionnaire used in both apps. Finally, a demo version of each app is accessible at https://bit.ly/main-app-demo and https://bit.ly/calibration-app-demo.

## Code availability

R code for the apps and all reported analyses is stored in the project’s OSF repository (https://osf.io/mhsr8/) which was accessible for peer-review, and is now public.
