# Author Correction: Understanding belief in political statements using a model-driven experimental approach: a registered report

**DOI:** 10.1038/s41598-024-52467-9

**Published:** 2024-01-23

**Authors:** Agustín Perez Santangelo, Guillermo Solovey

**Affiliations:** 1 Instituto de Investigación en Ciencias de la Computación, Universidad de Buenos Aires (UBA), Consejo Nacional de Investigaciones Científicas y Técnicas (CONICET), C1428EGA Buenos Aires, Argentina; 2 Laboratorio de Neurociencia, CONICET, Universidad Torcuato Di Tella, C1428BIJ Buenos Aires, Argentina; 3 Instituto de CálculoFacultad de Ciencias Exactas y Naturales, UBA-CONICET, Buenos Aires, Argentina

Correction to: *Scientific Reports* 10.1038/s41598-023-47939-3, published online 01 December 2023

The original version of this Article contained an error in the Abstract, which listed an incorrect direction of the association between cognitive reflection and partisan bias:

“Using Signal Detection Theory and Bayesian modeling, we found a reliable positive association between political concordance and overall belief in a statement (median = 0.663, CI95 = [0.640, 0.685]), a reliable positive association between cognitive reflection and scepticism (median = 0.039, CI95 = [0.006, 0.072]), a positive but unreliable association between cognitive reflection and truth discernment (median = 0.016, CI95 = [−0.015, 0.046]) and a negative but unreliable association between cognitive reflection and partisan bias (median = −0.016, CI95 = [−0.037, 0.006]).”

now reads:

“Using Signal Detection Theory and Bayesian modeling, we found a reliable positive association between political concordance and overall belief in a statement (median = 0.663, CI95 = [0.640, 0.685]), a reliable positive association between cognitive reflection and scepticism (median = 0.039, CI95 = [0.006, 0.072]), a positive but unreliable association between cognitive reflection and truth discernment (median = 0.016, CI95 = [−0.015, 0.046]) and a positive but unreliable association between cognitive reflection and partisan bias (median = 0.016, CI95 = [−0.006, 0.037]).”

The original Article has been corrected.

