# Supplementary material for: Understanding belief in political statements using a model-driven experimental approach: a registered report
Source: Sci Rep. 2023 Dec 1;13:21205. doi: 10.1038/s41598-023-47939-3 (PMC10692149; doi:10.1038/s41598-023-47939-3)
Supplement: Supplementary file 1 — Supplementary Information. [file 41598_2023_47939_MOESM1_ESM.pdf]

Supplementary Information to:

Understanding belief in political statements using a model-driven experimental approach: a registered report

Agustín Perez Santangelo[1,2,*], Guillermo Solovey[3,*]

[1] Instituto de Investigación en Ciencias de la Computación, Universidad de Buenos Aires (UBA), Consejo Nacional de Investigaciones Científicas y Técnicas (CONICET), C1428EGA Buenos Aires, Argentina

[2] Laboratorio de Neurociencia, CONICET, Universidad Torcuato Di Tella, C1428BIJ, Buenos Aires, Argentina

[3] Instituto de Cálculo, Facultad de Ciencias Exactas y Naturales, UBA-CONICET, Buenos Aires, Argentina

* Corresponding authors: Agustín Perez Santangelo (asantangelo@utdt.edu); Guillermo Solovey (gsolovey@gmail.com).

1. Parameter recovery

To demonstrate the feasibility of our methodological approach, we performed a parameter recovery analysis [Wilson2019] using fake data that we generated following the structure of the real data we will collect with the calibration and main apps (see Analysis plan). Below we describe the analysis pipeline, best understood when read alongside the corresponding R scripts in the project's OSF repository (https://osf.io/mhsr8/).

- For the calibration app analysis:
    1. We generated a design matrix for 1200 subjects, including all within and between-subjects variables that will be measured.
    2. We specified our multivariate zero-one inflated beta generalized linear mixed model (see Analysis plan, Equation 2).
    3. We fixed arbitrary —yet plausible— values for all model intercepts and for three coefficients associated with three arbitrary statements for each dependent variable. Importantly, we fixed these values by setting constant priors for each of these coefficients (henceforth, coefficients of interest).
    4. We sampled values for the model parameters using only the prior distributions (although for the coefficients of interest, this is actually a point value).
    5. We generated fake data (i.e., responses for the three dependent variables) using these sampled parameter values over the design matrix.
    6. We fit the fake data with the same model, although at this point —importantly— we specified weakly-informative priors for all coefficients, and used the fake data to sample the posterior distribution of the model parameters.
    7. We extracted the posterior distribution of each of the coefficients of interest.
    8. We assessed whether the fixed parameter values (used for generating fake data) were included in the 95-quantile credible interval of the posterior distribution of the corresponding coefficient of interest (Figure S4A).
    9. Finally, we computed the marginal (across political profiles, highest education level, and for participants' mean age) expected means for each statement for each dependent variable (ratings for pro-Macri and pro-Kirchner congruence) and we obtained the corresponding political valence values (i.e., pro-Macri congruence minus pro-Kirchner congruence rating means). These expected means were then saved to a dataframe which was later used for the parameter recovery analysis of the main app.

- For the main app analysis:
    1. We generated a design matrix for 1200 subjects, including all within and between-subjects variables that will be measured (which also includes the expected political valence means for the statements, obtained from the calibration app analysis).

2. We specified our hierarchical equal-variance Signal Detection Theory (SDT) model (see Analysis plan, Equation 1).
3. We set values for the coefficients that represent hypotheses H1, H2 and H4 (see Table 1) according to the effect sizes reported in a recent review studying belief in misinformation [18] (δ_{crt_score}= 0.45, $\lambda_{pol\_concord}$= -0.7, and λ_{crt_score}= 0.2), respectively, and at a conservative "small" effect size for H3 ($\lambda_{pol\_concord:crt\_score}$= 0.2). Additionally, we fixed arbitrary --yet plausible-- values for the model intercepts for the SDT parameters (*c* and *d'*). Importantly, we fixed all these values by setting constant priors for each of these coefficients (henceforth, coefficients of interest).
4. We sampled values for the model parameters using only the prior distributions (although for the coefficients of interest, this is actually a point value).
5. We generated fake data (i.e., true/false responses) using these sampled parameter values over the design matrix.
6. We fit the fake data with the same model, although now —importantly— we specified weakly informative priors for all coefficients, and used the fake data to sample the posterior distribution of the model parameters.
7. We extracted the posterior distribution of each of the coefficients of interest.
8. We assessed whether the fixed parameter values (used for generating fake data) were included in the 95-quantile credible interval of the posterior distribution of the corresponding coefficient of interest (Figure S4B). If it was included, then we interpreted this as a credible recovery of that coefficient.

Importantly, both parameter recovery analyses were successful, as evidenced by the inclusion of all the fixed parameter values within the credible intervals of the corresponding estimated coefficients' posterior distributions. This is strong support for the suitability of our analysis pipelines. We will run the same code (steps 6 and 8 for the main app analysis, and additionally step 9 for the calibration analysis) for the real collected data analysis pipeline (as detailed in the Analysis plan section).

2. Cognitive Reflection Test

3-item CRT in spanish and its original english version [Frederick2005]

Q1:

Una raqueta y una pelota de tenis cuestan $1100 en total. La raqueta cuesta $1000 más que la pelota. ¿Cuánto cuesta la pelota?

A bat and a ball cost $1.10 in total. The bat costs $1.00 more than the ball. How much does the ball cost?

[correct answer = $50 (*5 cents*); heuristic answer = $100 (*10 cents*)]

Q2:

Si a 5 máquinas les toma 5 minutos hacer 5 aparatos, ¿Cuánto tiempo (en minutos) le tomaría a 1000 máquinas hacer 1000 aparatos?

If it takes 5 machines 5 minutes to make 5 widgets, how long would it take 100 machines to make 100 widgets?

[correct answer = 5 minutos (*5 minutes*); heuristic answer = 1000 minutos (*100 minutes*)]

Q3:

En un lago hay una mancha de aceite. Cada día, la mancha duplica su tamaño. Si toma 48 días para que la mancha cubra el lago completamente, ¿Cuántos días tomaría para que la mancha cubra la mitad del lago?

In a lake, there is a patch of lily pads. Every day, the patch doubles in size. If it takes 48 days for the patch to cover the entire lake, how long would it take for the patch to cover half of the lake?

[correct answer = 47 días (*47 days*); heuristic answer = 24 días (*24 days*)]

3. Assessment of numeracy

Three questions in spanish and their english original version [Schwartz1997].

Q1:

Imagine que tira una moneda 1000 veces. ¿En cuántas de esas 1000 veces cree que la moneda saldría cara?

Imagine that we flip a fair coin 1,000 times. What is your best guess about how many times the coin would come up heads in 1,000 flips?

[correct answer = 500]

Q2:

En una lotería, la posibilidad de ganar un premio de $10 es del 1%. ¿Cuántas personas cree que ganarían un premio de $10 si 1000 personas compran -cada una- un solo billete de la lotería?

In the BIG BUCKS LOTTERY, the chance of winning a $10 prize is 1%. What is your best guess about how many people would win a $10 prize if 1000 people each buy a single ticket to BIG BUCKS?

[correct answer = 10]

Q3:

En una lotería, la posibilidad de ganar un auto es de 1 en 1000. ¿Qué porcentaje de billetes de la lotería tienen un billete ganador del auto?

In ACME PUBLISHING SWEEPSTAKES, the chance of winning a car is 1 in 1,000. What percent of tickets to ACME PUBLISHING SWEEPSTAKES win a car?

[correct answer = 0.1 %]

Figure S1. Demographic variables of participants of the main study (N=1353)

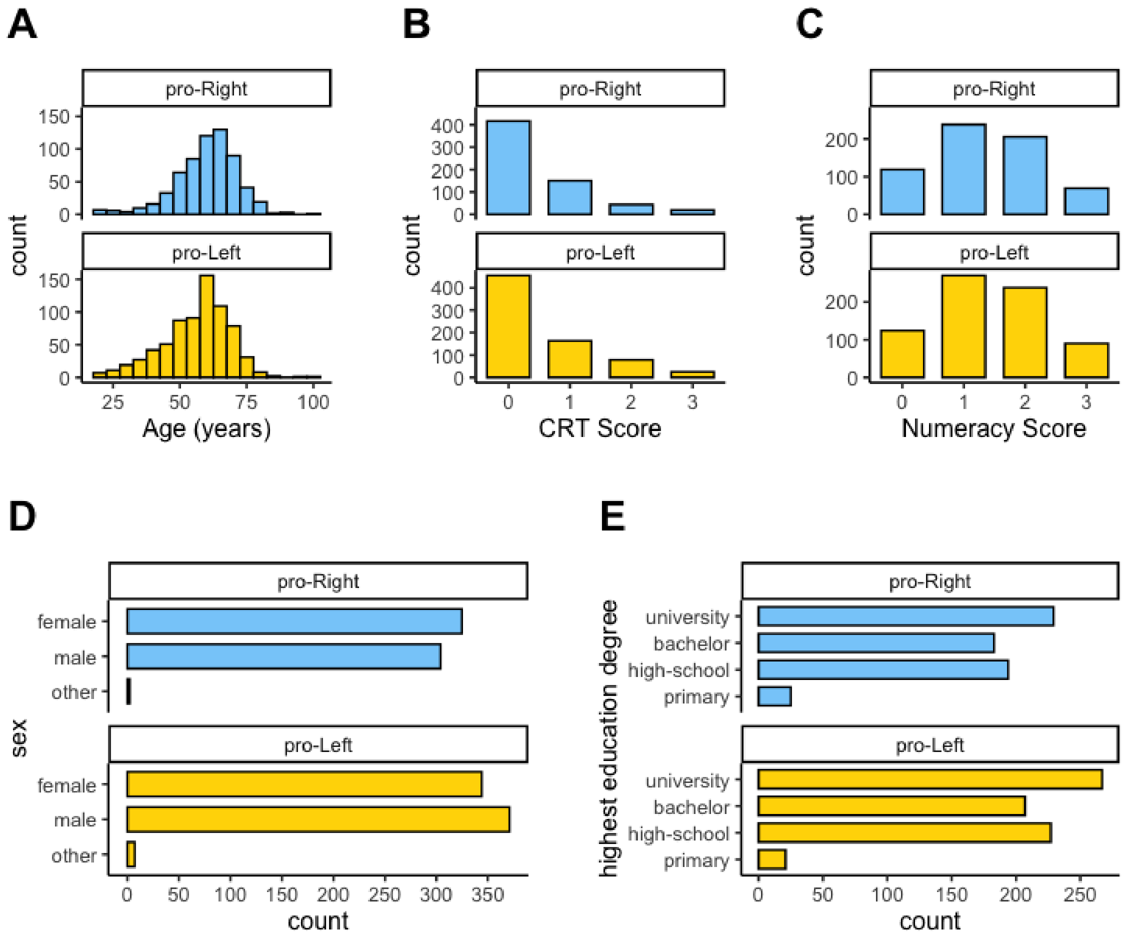

Figure S2. Demographic variables of participants of the calibration task (N=1210)

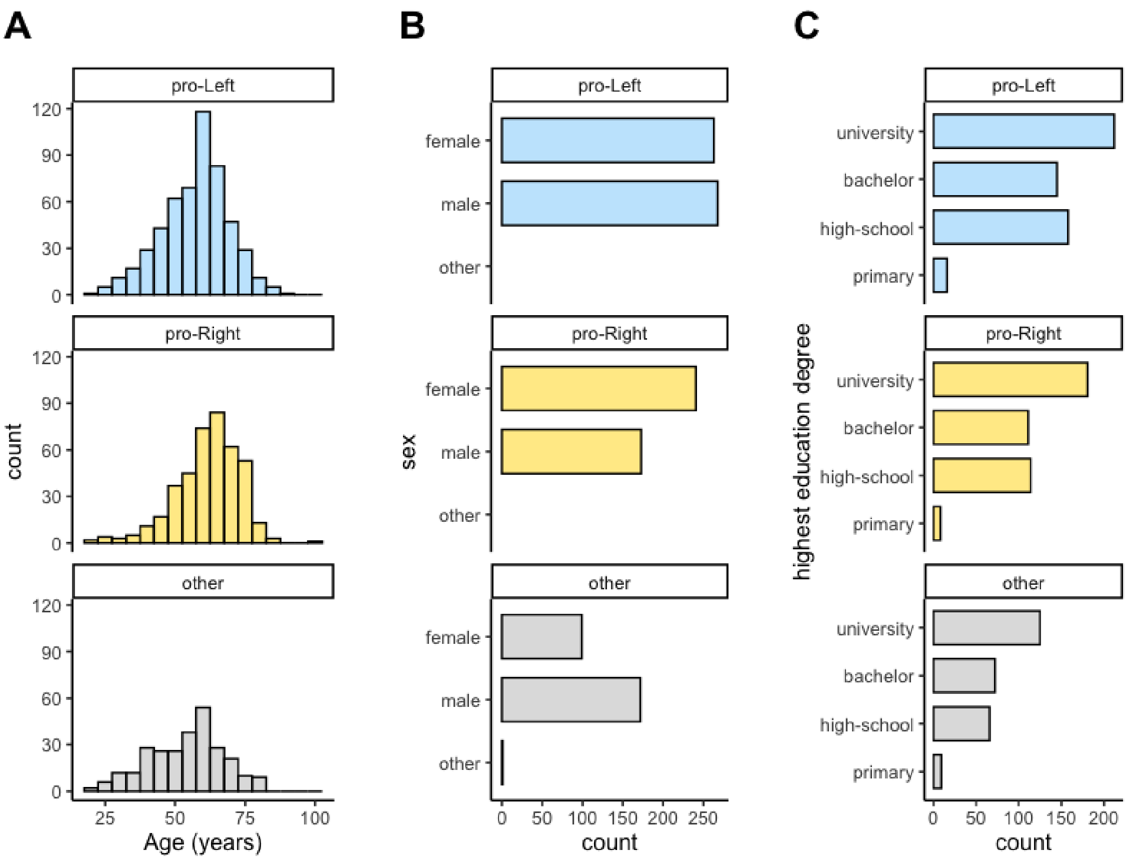

Figure S3. pro-Left and pro-Right congruence of each statement

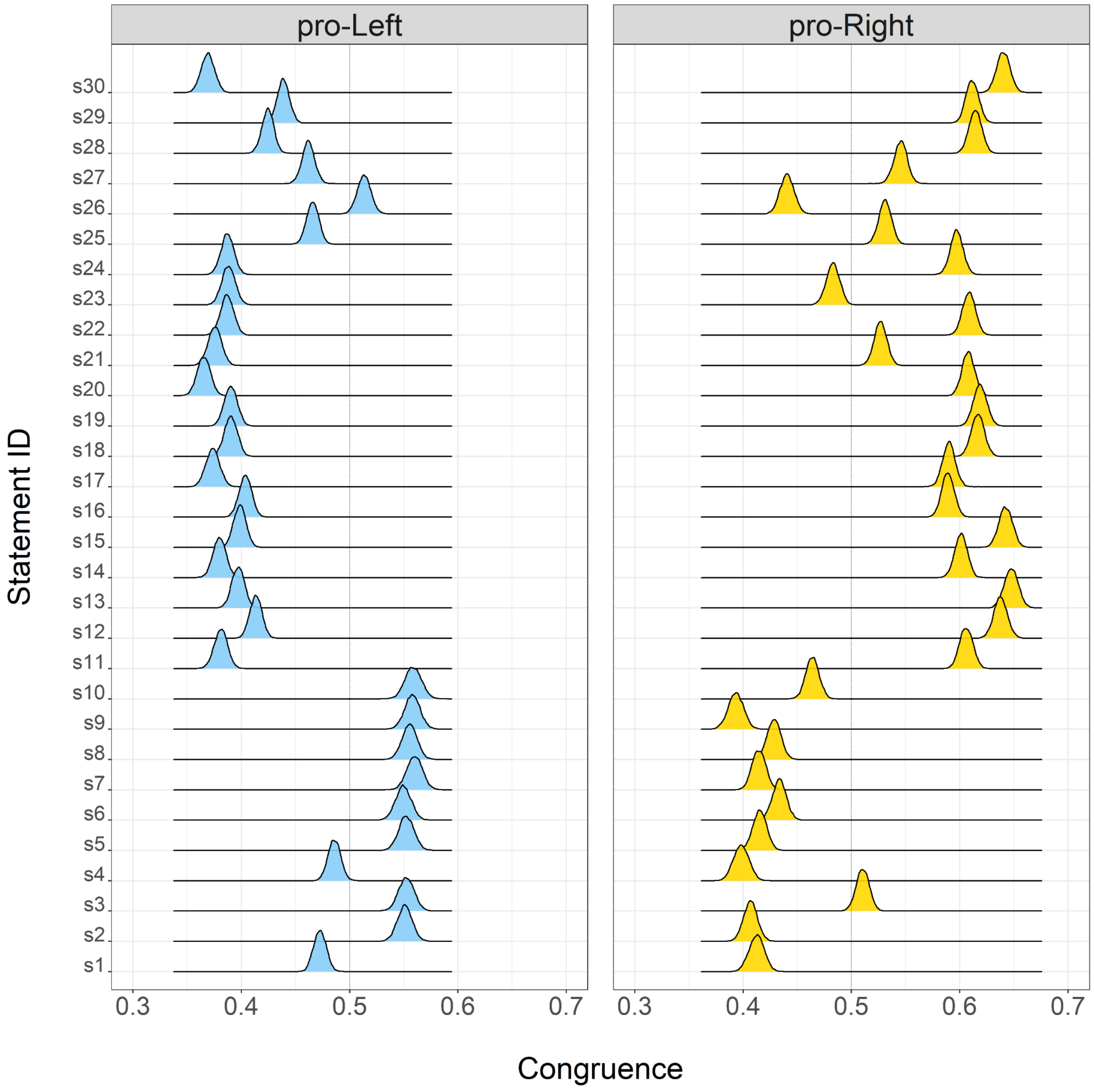

Figure S3. pro-Left and pro-Right congruence of each statement. Statements 1 to 10 were made by a politician from the pro-Left coalition, statements 11 to 20 were made by a politician from the pro-Right coalition, and statements 21 to 30 were made by a politician from neither the pro-Left nor pro-Right coalitions.

Figure S4. Parameter recovery analysis.

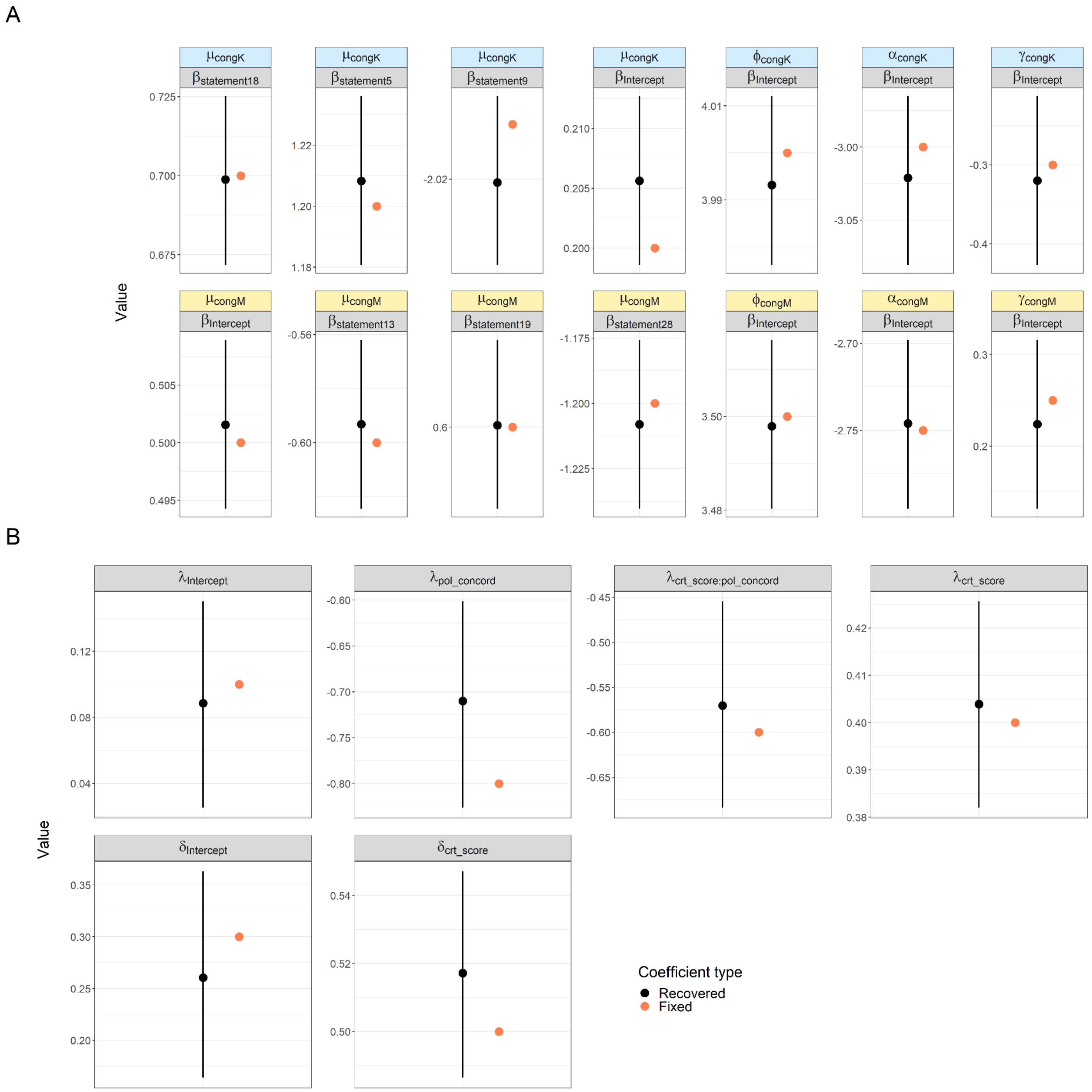

Figure S4. Parameter recovery analysis. Results of the parameter recovery for the main and calibration analyses (described in full in the Supplementary Information section). This procedure aims to assess whether fixed (i.e., known) model coefficients' values can be recovered by fitting our models with fake data generated with these fixed values. Fake data has the same structure as real collected data will

have. A. For the calibration analysis, all coefficients were successfully recovered across dependent variables (*congK* (light blue), *congM* (light yellow)) and distributional parameters (μ, ϕ, α and γ), as all fixed values (light red points) are contained within the 95% credible interval of the corresponding recovered posterior values (black vertical lines), in some cases very close to the median of the recovered posterior values (black dot). B. Similarly, for the main analysis, all coefficients of interest (see Table 1) were successfully recovered. Since both recovery analyses were successful, we can be confident that the proposed analytical strategy (see Analysis plan) is feasible and reliable.

Figure S5. Summary of estimated model coefficients.

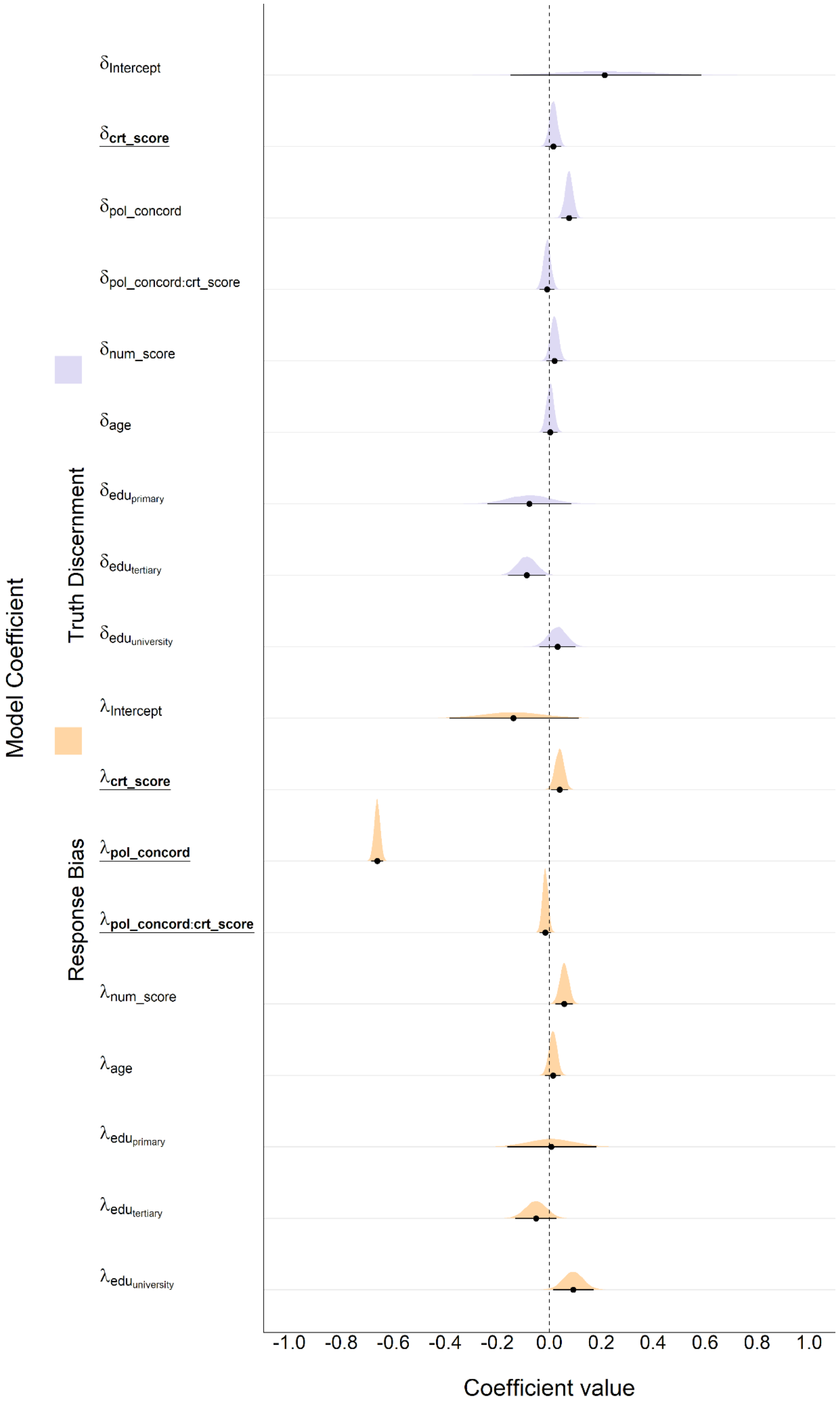

Table S1. Corpus of 30 statements made by political leaders and elected officials in Argentina. Statements were previously fact-checked by Chequeado (www.chequeado.com) as either true or false. Each statement was presented to participants as detailed in the main text and Figures 3 and 4.

ID	statement	fact-check
s1	Alberto Fernández (Presidente de la Nación):<br>*“900% de inflación tuvo Estados Unidos”*. Septiembre, 2022.	F
s2	Alberto Fernández (Presidente de la Nación):<br>*“En los 4 años previos a nuestra llegada, la producción de gas había caído a un ritmo del 8% anual”*. Agosto, 2022.	F
s3	Alberto Fernández (Presidente de la Nación):<br>*“Ningún magistrado ni funcionario judicial paga hoy el impuesto a las Ganancias”*. Marzo, 2021.	F
s4	Alberto Fernández (Presidente de la Nación):<br>*“En 2021 la pobreza dejó de aumentar”*. Marzo, 2022.	T
s5	Alberto Fernández (Presidente de la Nación):<br>*“Construimos 12 hospitales modulares en tiempo récord”*. Marzo, 2021.	T
s6	Victoria Tolosa Paz (Ministra de Desarrollo Social de la Nación):<br>*“Vidal, en 4 años, ni una cárcel construyó”*. Octubre, 2021.	F
s7	Victoria Tolosa Paz (Ministra de Desarrollo Social de la Nación):<br>*“La Ciudad que gobierna Santilli ajusta año tras año el presupuesto de Educación”*. Octubre, 2021.	T
s8	Santiago Cafiero (ex Jefe de Gabinete de Ministros de la Nación):<br>*“La Argentina es uno de los 12 países en el mundo que produce vacunas”*. Junio, 2021.	T
s9	Gabriela Cerruti (Portavoz de la Presidencia de la Nación):<br>*“No hay ninguna instancia judicial en que la Justicia argentina diga que el fiscal Nisman fue asesinado”*. Agosto, 2022.	F
s10	Martín Guzmán (ex Ministro de Economía de la Nación):<br>*“Esos casi US$ 45 mil millones es una fortuna enorme, récord histórico en la historia del FMI” (sobre el préstamo otorgado en 2018)*. Enero, 2022.	T

s11	María Eugenia Vidal (Diputada de la Nación por la Provincia de Buenos Aires):<br>*“Marcos Peña fue todos los meses a rendir cuentas” al Congreso como jefe de Gabinete*. Febrero, 2022.	F
s12	María Eugenia Vidal (Diputada de la Nación por la Provincia de Buenos Aires): Sobre el gobierno de Alberto Fernandez: *“El salario perdió 20 puntos y las jubilaciones 11”*. Octubre, 2021.	F
s13	María Eugenia Vidal (Diputada de la Nación por la Provincia de Buenos Aires):<br>*“Estamos peor que en diciembre de 2019. Hay más pobreza”.* Octubre, 2021.	T
s14	Horacio Rodríguez Larreta (Jefe de Gobierno de la Ciudad de Buenos Aires):<br>*“Sumamos 110 hectáreas de parques nuevos”*. Septiembre, 2021.	F
s15	Horacio Rodríguez Larreta (Jefe de Gobierno de la Ciudad de Buenos Aires):<br>*“Hoy un chico puede votar a los 16 años pero no es responsable. No puede ser que no sea punible”*. Noviembre, 2021.	F
s16	Horacio Rodríguez Larreta (Jefe de Gobierno de la Ciudad de Buenos Aires):<br>*“Se bajó la tasa de desempleo femenino del 13,4% al 9,5%”.* Marzo, 2022.	T
s17	María Soledad Acuña (Ministra de Educación de la Ciudad de Buenos Aires).<br>*Sobre el presupuesto educativo de la Ciudad: “Es el que más aumentó en 2021 respecto a otras áreas”*. Mayo, 2021.	F
s18	Martín Tetaz (Diputado de la Nación por la Ciudad de Buenos Aires):<br>*“Hace 11 años que en la Argentina no se crea empleo privado”.* Junio, 2022.	T
s19	Mauricio Macri (ex Presidente de la Nación):<br>*“Nuestra producción de granos está estancada”*. Julio, 2022.	T
s20	Diego Santilli (Diputado de la Nación por la Provincia de Buenos Aires):<br>*“Cuando me tocó trabajar en Seguridad bajamos todos los delitos”.* Octubre, 2021.	T
s21	Javier Milei (Diputado de la Nación por la Ciudad de Buenos Aires):<br>*“Aquellos Estados que tienen libre portación de armas tienen*	F

	muchos menos delitos". Junio, 2022.	
s22	Javier Milei (Diputado de la Nación por la Ciudad de Buenos Aires):<br>*"Los indicadores sociales de la economía son peores que los que teníamos en 2001"*. Agosto, 2022.	F
s23	Javier Milei (Diputado de la Nación por la Ciudad de Buenos Aires):<br>*"El calentamiento global es una mentira"*. Agosto, 2021	F
s24	Javier Milei (Diputado de la Nación por la Ciudad de Buenos Aires):<br>*"Todas las empresas (del Estado) tienen un déficit del 0,7% del PBI"*. Abril, 2022.	T
s25	Nicolás Del Caño (Diputado de la Nación por la Provincia de Buenos Aires):<br>*"Los intendentes de Juntos y del Frente de Todos ganan entre $ 600 mil y $ 1,2 millones"*. Octubre, 2021.	F
s26	Francisco Meritello (ex Secretario de Medios de la Nación en el período 2019-2021): Sobre el gobierno de Alberto Fernandez:<br>*"En un año y 4 meses no hemos tenido una sola denuncia de corrupción"*. Marzo, 2021.	F
s27	Myriam Bregman (Diputada de la Nación por la Ciudad de Buenos Aires):<br>*"Manzur obligó a una niña de 11 años violada a parir"*. Octubre, 2021.	T
s28	Martín Lousteau (Senador de la Nación por la Ciudad de Buenos Aires):<br>*"Cada vez que en la Argentina la inflación nos come un 1% del poder adquisitivo hay 230 mil pobres nuevos".* Mayo, 2022.	T
s29	José Luis Espert (Diputado de la Nación por la Ciudad de Buenos Aires):<br>*"4 millones [de personas] viven en villas miseria"*. Octubre, 2021.	T
s30	Guido Sandleris (ex Presidente del Banco Central):<br>*"Durante los gobiernos de CFK el resultado fiscal empeoró todos los años"*. Abril, 2021.	T

References

[Frederick2005] Frederick, S. Cognitive Reflection and Decision Making. J. Econ. Perspect. 19,25–42 (2005).

[Schwartz1997] Schwartz, L. M., Woloshin, S., Black, W. C., & Welch, H. G. (1997). The role of numeracy in understanding the benefit of screening mammography. Annals of internal medicine, 127(11), 966-972.

[Wilson2019] Wilson, R. C. & Collins, A. G. Ten simple rules for the computational modeling of behavioral data. eLife 8, (2019).
